# Chitosan-Coated Oleuropein Nanoliposomes Enable Dose-Sparing Suppression of Serum Inflammatory Markers in a TNF-α-Induced Vascular Inflammation Model

**DOI:** 10.1021/acsomega.6c06263

**Published:** 2026-09-23

**Authors:** Roi Martin B. Pajimna, John Wilfred T. Malabanan, Nonthaneth Nalinratana, Yilin Song, Pranee Rojsitthisak, Pornchai Rojsitthisak, Agnes L. Llamasares-Castillo

**Affiliations:** † The Graduate School, 595549 University of Santo Tomas, Manila 1015, Philippines; ‡ Department of Pharmacy, Faculty of Pharmacy, University of Santo Tomas, Manila 1015, Philippines; § Center of Excellence in Natural Products for Ageing and Chronic Diseases, 26683 Chulalongkorn University, Bangkok 10330, Thailand; ∥ Department of Pharmacy, College of Allied Health Sciences, Trinity University of Asia, Quezon City 1102, Philippines; ⊥ Department of Pharmacology and Physiology, Faculty of Pharmaceutical Sciences, Chulalongkorn University, Bangkok 10330, Thailand; # Department of Pharmaceutics, School of Pharmacy, 58575 Shenyang Pharmaceutical University, Shenyang, Liaoning 110016, China; ∇ Metallurgy and Materials Science Research Institute, Chulalongkorn University, Bangkok 10330, Thailand; ○ Department of Food and Pharmaceutical Chemistry, Faculty of Pharmaceutical Sciences, Chulalongkorn University, Bangkok 10330, Thailand; ◆ Research Center for the Natural and Applied Sciences, University of Santo Tomas, Manila 1015, Philippines

## Abstract

Vascular inflammation, marked by endothelial dysfunction and overexpression of proinflammatory cytokines such as interleukin-6 (IL-6) and monocyte chemoattractant protein-1 (MCP-1), is a key contributor to atherosclerosis. Oleuropein (Ole), a phenolic compound with proven anti-inflammatory properties, suffers from poor aqueous stability and limited oral bioavailability, constraining its clinical application. To overcome these limitations, we developed and optimized chitosan (CS)-coated liposomes for oral delivery of Ole. Using a Box-Behnken design, the optimized formulation exhibited a particle size of 168.73 ± 3.86 nm, a polydispersity index (PDI) of 0.17 ± 0.01, a moderate negative zeta potential (−22.50 ± 2.39 mV), an encapsulation efficiency of 87.17 ± 0.27%, and a loading capacity of 12.22 ± 0.25%. The CS-coated formulation retained acceptable colloidal stability under refrigerated storage, enabled pH-responsive sustained release governed by Fickian diffusion, and enhanced endothelial nanocarrier uptake in EA.hy926 cells. The formulation was well tolerated in vitro, with cell viability exceeding 90% across a 500–4000 ng/mL concentration range. In a TNF-α-induced murine model of acute vascular inflammation, daily oral administration of CS-Ole-Lip (20 mg/kg) significantly suppressed serum IL-6 by 6.90-fold (*p* < 0.05) and MCP-1 by 2.45-fold relative to TNF-α controls (*p* < 0.0001), with numerically greater suppression than that observed with free Ole and uncoated Ole-Lip at the same dose. CS-Ole-Lip at 2 mg/kg, one-tenth of the 20 mg/kg dose evaluated in this study, produced substantial serum cytokine suppression, suggesting a potential dose-sparing effect. No treatment-associated elevations in the evaluated serum markers of hepatic or renal function or overt histological abnormalities in the examined organs were observed. These findings support CS-Ole-Lip as a stable oral nanocarrier with enhanced anti-inflammatory pharmacodynamic activity. The mechanistic basis for the dose-sparing effect requires direct pharmacokinetic and molecular validation.

## Graphical abstract

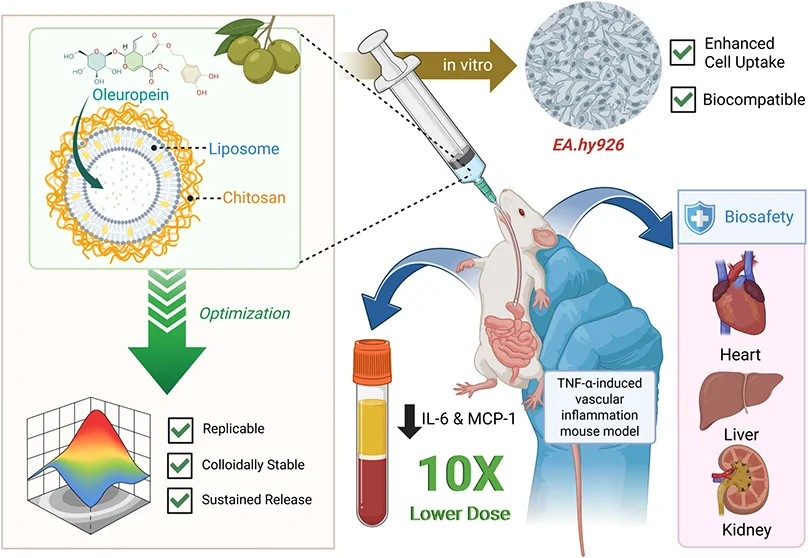

## 1 Introduction

Vascular inflammation is a central pathophysiological process in the development and progression of numerous cardiovascular diseases (CVD), including atherosclerosis, hypertension, and vascular complications of metabolic diseases.^1,2^ Endothelial activation promotes barrier disruption, oxidative stress, and a proinflammatory secretome that includes interleukin-6 (IL-6) and monocyte chemoattractant protein-1 (MCP-1), thereby programming leukocyte recruitment and amplifying downstream inflammatory cascades.^3,4^ Despite the causal role of endothelial inflammation, therapeutic development in CVD has remained dominated by lipid lowering and systemic immunomodulation, leaving a persisting need for interventions that directly and durably attenuate endothelial inflammatory signaling at the vascular interface.

Oleuropein (Ole), a phenolic secoiridoid abundant in olive leaves and unrefined olive oil, has been reported to mitigate endothelial inflammatory activation by suppressing nuclear factor kappa B (NF-κB) signaling, thereby reducing TNF-α-induced cytokine production and the expression of adhesion-related mediators.^5,6^ However, the translational trajectory of Ole remains constrained by chemical instability and low oral bioavailability, which reduce the fraction of intact compound available for vascular exposure and generate inconsistent pharmacodynamic readouts.^7,8^ An enabling delivery strategy must therefore address both compound integrity and exposure control to realize reproducible anti-inflammatory activity.

Nanoliposomal encapsulation provides a versatile platform in which bilayer composition, surface structure, and interfacial chemistry can be modified to protect labile polyphenols, modulate release kinetics, and direct biological interactions at the endothelial interface.^9–11^ Yet conventional liposomes are prone to leakage, aggregation, and rapid clearance, particularly under electrolyte-rich or protein-containing conditions that challenge bilayer integrity and colloidal stability.^12,13^ Surface functionalization with biopolymers provides a mechanistically grounded approach to overcome these limitations by stabilizing the interface and controlling cargo retention during transit across gastrointestinal and physiological environments. Chitosan (CS) is a cationic polysaccharide that has been the subject of GRAS notices for specific food uses and is described in pharmacopeial monographs; these regulatory contexts do not constitute US FDA approval as a pharmaceutical excipient for oral drug products.^14–16^ CS exhibits pH-responsive solubility and mucoadhesive behavior, enabling prolonged intestinal residence and electrostatic association with negatively charged biological surfaces. These features position CS coating as a promising strategy to strengthen liposome stability and promote prolonged gastrointestinal residence, which may improve the likelihood of maintaining an intact payload during transit.

Several CS-Lip systems have been reported for other therapeutic contexts: CS-Lip coloaded with exemestane and genistein was developed for combination breast cancer chemotherapy without response surface optimization;^17^ CS-coated curcumin liposomes were stabilized against degradation using high-pressure processing to improve bioactivity retention;^18^ and resveratrol-loaded chitosan nanoparticles were engineered for anti-*Helicobacter pylori* activity via a gastric-targeted delivery route.^19^ To our knowledge, no previously reported CS-Lip polyphenol system has been evaluated in a TNF-α-induced vascular inflammation model with a quantified pharmacodynamic dose-sparing factor, nor optimized using response surface methodology for this application. Related precedents include a Box-Behnken-optimized, chitosan-coated liposomal system developed for a nonpolyphenol anticancer agent,^20^ design of experiments (DoE)-optimized chitosan-based polyphenol nanocarriers evaluated in nonvascular inflammatory models,^21,22^ and chitosan nanoparticle-encapsulated oleuropein, which achieved a comparable dose-sparing pattern in a gastroprotective, nonvascular model.^23^ These studies collectively underscore the distinct contribution of the present BBD-optimized CS-Ole-Lip system for TNF-α-induced vascular inflammation. In this context, we hypothesized that surface modification with CS would increase Ole stability and retention within nanoliposomes across gastrointestinal and physiological environments, thereby improving dose integrity and enhancing suppression of TNF-α-driven endothelial inflammatory activation relative to uncoated Ole-loaded liposomes (Ole-Lip) and free Ole. To test this hypothesis, we developed Ole-Lip with and without CS coating and optimized key formulation variables using Box-Behnken design (BBD). We then established a relationship between formulation attributes and biological outcomes by assessing physicochemical performance, Ole retention and release across biorelevant media, and anti-inflammatory efficacy in a biological system. Therapeutic activity and systemic safety were further examined in a TNF-α-induced acute vascular inflammation mouse model, supported by serum biochemical indices (ALT, AST, BUN, creatinine) and histopathological evaluation.

## 2 Experimental Section

### 2.1 Materials and Reagents

Ole (98% purity) was purchased from BLD Pharm, Shanghai, China. Soybean lecithin (90% purity) was obtained from Thermo Fisher Scientific Chemicals, Inc., Eindhoven, Netherlands, while cholesterol (>95% purity) was sourced from Tokyo Chemical Industry Co., Ltd., Tokyo, Japan. Chitosan (CS), with a molecular weight of approximately 540 kDa and a degree of deacetylation greater than 90%, was obtained from Marine Bio-Resources Co., Ltd., Samut Prakan, Thailand. EA.hy926 endothelial cells were acquired from the American Type Culture Collection (ATCC), Manassas, VA, USA. Dulbecco’s Modified Eagle’s Medium (DMEM), fetal bovine serum (FBS), penicillin (100 U/mL), and streptomycin (100 μg/mL) were purchased from Gibco, Thermo Fisher Scientific, Waltham, MA, USA. MTT solution was obtained from Sigma-Aldrich, St. Louis, MO, USA. All other reagents and solvents used were of at least analytical grade and employed without further purification.

### 2.2 Preparation of CS-Ole-Lip

The preparation of CS-Ole-Lip was carried out according to previously established protocols with minor modifications.^24,25^ The preweighed soy lecithin and cholesterol were dissolved in 5 mL chloroform and were subjected to rotary evaporation at 40 °C, 120 rpm, 200 mbar (G3 Heidolph Hei-VAP Core, Heidolph Instruments GmbH & Co. KG, Bavaria, Germany) to form the thin lipid film (Figure 1). The flask was kept in a desiccator overnight to ensure full removal of chloroform via evaporation. Subsequently, the lipid film was detached from the flask wall and rehydrated in a rotary evaporator at 55 °C and 120 rpm for 15 min using 3 g of glass beads (diameter: 3 mm) and 10 mL of phosphate-buffered saline (PBS, pH 7.4) containing Ole at a known concentration. The resulting liposome suspension was subjected to high-pressure homogenization using a high-shear benchtop homogenizer (LV1 Microfluidizer, Microfluidics Corp., Westwood, MA, USA) at 500 bar for one passage.

**Figure 1:**
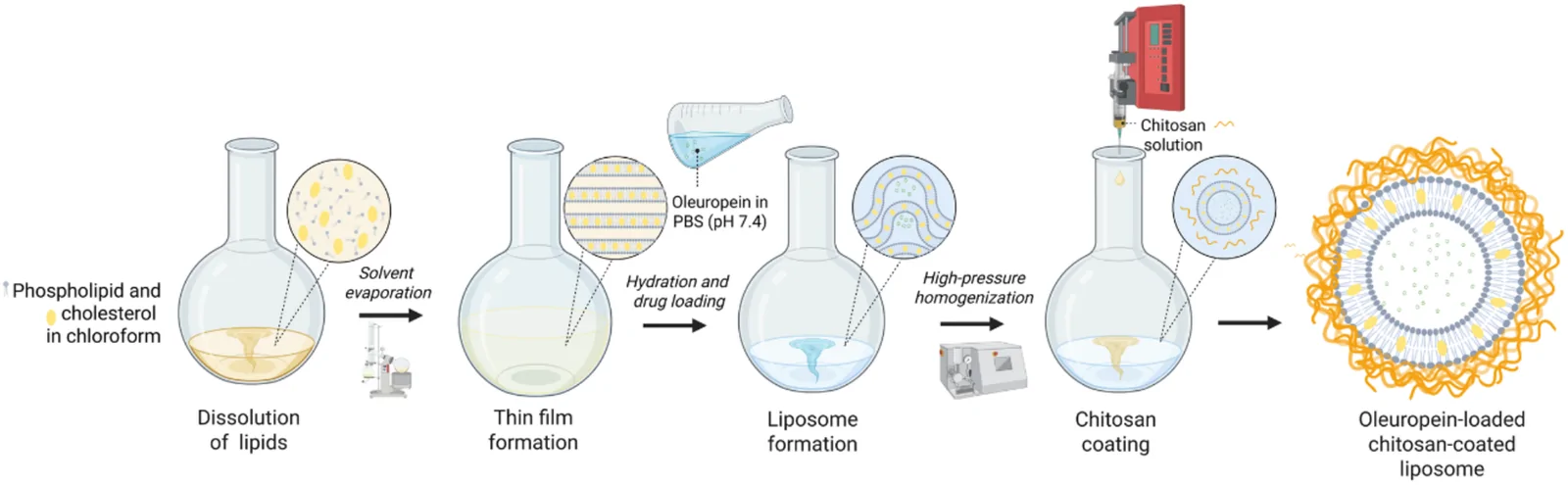
Schematic representation of the preparation of CS-Ole-Lip.

After homogenization, 10 mL of a CS solution of known concentration in 1% *(v/v)* acetic acid was added dropwise at a rate of 40 mL/h using an automatic syringe pump (NE-1000 Single Syringe Pump, New Era Pump Systems, Inc., Farmingdale, NY, USA) under continuous stirring at 1200 rpm (MS-H380-Pro Hot plate Magnetic Stirrer, Onilab LLC Scientific Inc., Riverside, CA, USA). Stirring was continued for an additional 1 h to ensure uniform surface coating. The formulation was then equilibrated at 4 °C overnight to ensure stabilization prior to analysis. A blank CS-coated liposome formulation was prepared following the same procedure, excluding Ole.

### 2.3 Optimization Using BBD

The experiment was focused on three critical formulation factors: amount of Ole (*A*), lecithin/cholesterol mass ratio (*B*), and CS concentration (*C*). To evaluate the effects of these variables on particle size (*Y*_1_), polydispersity index (PDI) (*Y*_2_), and loading capacity (LC) (*Y*_3_) of the CS-Ole-Lip, a three-factor, three-level BBD was employed as summarized in Table 1. A total of 15 experimental runs were generated using Design-Expert software (Version 13, Stat-Ease Inc., Minneapolis, MN, USA). Each run was performed in triplicate and conducted in randomized order.

**Table 1:** Variables and Levels of the BBD

Variables	Levels		
Independent (Factors)	Low (−1)	Medium (0)	High (+1)
*A*: Amount of Ole (mg)	10	15	20
*B*: Lecithin/Cholesterol Mass Ratio	2:1	3.5:1	5:1
*C*: CS Concentration (mg/mL)	0.05	0.075	0.10
*Dependent (Responses)*	*Constraints*		
*Y*_1_ = Particle size (nm)	Minimize		
*Y*_2_ = PDI	Minimize		
*Y*_3_ = LC (%)	Maximize		

Analysis of variance (ANOVA) was carried out to determine the statistical significance of the model terms (Table S1). Response surface plots were used to visualize the interaction effects among variables and to identify the optimal formulation conditions with desirability closest to 1.00. In the numerical optimization, *Y*_1_ (particle size) and *Y*_2_ (PDI) were set to minimize, whereas *Y*_3_ (loading capacity) was set to maximize; importance levels of +++++, ++++, and +++++ were assigned to *Y*_1_, *Y*_2_, and *Y*_3_, respectively, and default lower and upper weight values of 1 were retained for all responses. The optimized formulation was then prepared and experimentally validated, and the predicted values for the selected responses were confirmed. Model robustness was further evaluated by preparing the optimized formulation in six independent replicates. In addition to the 95% prediction intervals (PI), the percentage error for each response was calculated using eq 1.

$$\%error=\frac{\left(observed\;value-predicted\;value\right)}{\left(predicted\;value\right)}\times 100$$ 1

### 2.4 Physicochemical Characterization of CS-Ole-Lip

The particle size, PDI, and zeta (ζ) potential of the CS-Ole-Lip were measured using a DLS instrument (Zetasizer Nano ZS, Malvern Instruments Ltd., Worcestershire, U.K.) at 25 °C. Samples were gently equilibrated for 10 s and measured in triplicate. Encapsulation efficiency (EE) was determined indirectly by ultracentrifugation (45,000 rpm for 1 h at 4 °C). Free Ole in the supernatant was collected and quantified via ultraviolet–visible (UV–Vis) spectrophotometry (Agilent Cary 60, Santa Clara, CA, USA) at 281 nm using a PBS (pH 7.4) calibration curve (*y* = 0.0234*x* + 0.0184). Potential spectral interference from lipid and CS components was excluded by confirming negligible absorbance at 281 nm in supernatants obtained from blank CS-coated liposomes (CS-Lip) processed under identical ultracentrifugation conditions. The EE and LC were calculated using eqs 2 and 3, respectively.

2 $$EE\;\left(\%\right)=\frac{amount\;of\;Ole\;initially\;added-amount\;of\;Ole\;in\;the\;superna\tan t}{amount\;of\;Ole\;initially\;added}\times 100$$

$$LC\;\left(\%\right)=\frac{amount\;of\;Ole\;initially\;added-amount\;of\;Ole\;in\;the\;superna\tan t}{weight\;of\;dried\;\mathrm{CS‐Ole‐Lip}}\times 100$$ 3

The morphology of the nanoparticles was examined via transmission electron microscopy (TEM; JEM-1400, JEOL Ltd., Tokyo, Japan) using 1% uranyl acetate solution as the negative stain. For TEM grid preparation, 10 μL of the CS-Ole-Lip dispersion was placed onto a copper grid and allowed to stand for 5 min to permit sample adsorption. Excess sample was gently blotted from the edge of the grid using filter paper. The grid was then negatively stained by adding 10 μL of 1% uranyl acetate solution and allowing it to stand for 2 min. Excess stain was carefully removed using filter paper, and the grid was left to dry overnight at room temperature prior to TEM imaging. The sample was prepared directly from the PBS-based dispersion without prior dialysis or buffer exchange. To compare the surface functional groups of CS-Ole-Lip with those of Ole, lecithin, cholesterol, and CS, Fourier-transform infrared (FTIR) spectroscopy was conducted using a Spectrum One FTIR spectrometer (PerkinElmer Inc., Waltham, MA, USA) in the 400–4000 cm^–1^ range. Furthermore, the crystalline structure of the nanoparticles was analyzed using powder X-ray diffraction (XRD; PANalytical X’Pert Pro, Kassel-Waldau, Germany) operated at 40 kV and 40 mA, scanned in continuous mode at a step size of 0.02° 2θ at 25 °C.

### 2.5 Colloidal Stability Studies

The colloidal stability of the optimized CS-Ole-Lip suspension was evaluated under refrigerated (4.0 ± 2.0 °C) and room temperature (RT; 25.0 ± 2.0 °C) storage conditions for a period of three months. At predetermined intervals, samples were collected to assess changes in particle size, zeta potential and EE.

### 2.6 In Vitro Release Study

Ole release from CS-Ole-Lip was evaluated in enzyme-free simulated gastric fluid (SGF, pH 1.2), simulated intestinal fluid (SIF, pH 6.8), and physiological buffer (pH 7.4) to mimic blood conditions, with each medium supplemented with 0.1% (w/v) Tween 80 to promote sink conditions. A 4-mL aliquot of CS-Ole-Lip suspension was loaded into SnakeSkin dialysis tubing (MWCO 3500 Da; Thermo Fisher Scientific, Waltham, MA, USA) and immersed in 40 mL of the corresponding release medium maintained at 37 °C under continuous agitation at 150 rpm. At predefined time points, 2 mL aliquots were collected from the external medium and immediately replaced with an equal volume of fresh, prewarmed medium to maintain constant volume. Ole in the collected samples was quantified by UV–Vis spectrophotometry at 281 nm, and cumulative release profiles were constructed as a function of time. The cumulative percentage release was calculated as follows

4 $$\mathrm{CR}\;\left(\%\right)=\frac{V_{e}\sum_{i=1}^{n-1}C_{n-1}+V_{o}C_{n}}{m}\times 100$$

where CR is cumulative release percentage, *V*_e_ is collection volume (mL), *V*_o_ is the volume of release medium (mL), *C_n_* is the drug concentration at the corresponding time point (mg/mL), and *m* is total Ole content (mg).^26^

Release kinetics were further analyzed by fitting the data in various kinetic models using the DDSolver add-in program in Microsoft Excel 365. The best-fit model was identified based on the goodness-of-fit test using the highest correlation coefficient, lowest Akaike information criterion (AIC), and highest model selection criterion (MSC).^27^

### 2.7 In Vitro Cellular Uptake Study

The cellular internalization of CS-Lip was qualitatively assessed by confocal laser scanning microscopy (CLSM; LSM 980 with Airyscan 2, Carl Zeiss, Jena, Germany) using a previously reported method with minor modifications.^27^ For fluorescence tracing, FITC was used as a model fluorescent probe in place of Ole to enable visualization of liposome internalization. FITC-loaded CS-Lip was prepared by substituting Ole with FITC using the procedure described in Section 2.2. It is acknowledged that FITC differs from Ole in molecular weight, hydrophobicity, and charge, and therefore the encapsulation behavior and intracellular distribution of FITC may not fully replicate those of Ole. Nevertheless, FITC loading serves as a well-established qualitative indicator of nanocarrier uptake. EA.hy926 cells were seeded to ∼50% confluence and incubated for 4 h with either free FITC or FITC-loaded CS-Lip. Cells treated with serum-free medium served as the negative control. Following incubation, cells were washed three times with cold PBS, fixed with 4% (w/v) paraformaldehyde, and imaged by CLSM.

### 2.8 In Vitro Cytocompatibility Studies

#### 2.8.1 Cell Culture

Human endothelial EA.hy926 (American Type Culture Collection, ATCC CRL-2922, passages 23–25) cells were cultured in DMEM, supplemented with 10% FBS, 100 U/mL penicillin, and 100 μg/mL streptomycin. The cells were maintained in a humidified atmosphere with 5% CO_2_ at 37 °C (Forma Series 3 Water Jacketed CO_2_ Incubator, Thermo Fisher Scientific, Waltham, MA, USA).

#### 2.8.2 MTT Cytocompatibility Assay

EA.hy926 cells were seeded into a 96-well plate at a density of 2 × 10^5^ cells/mL and incubated for 24 h at 37 °C. Then, the cells were treated with varying concentrations of CS-Lip, CS-Ole-Lip, or free Ole and were incubated for 24 h at 37 °C. After the treatment period, the medium was carefully removed, and 100 μL of MTT solution (0.5 mg/mL) was added to each well. The cells were incubated for 2 h at 37 °C to allow for formazan formation. The MTT solution was then discarded, and the resulting formazan crystals were dissolved in 100 μL of DMSO. Absorbance was recorded at 570 nm using a microplate reader (CLARIOstar Plus, BMG Labtech, Ortenberg, Germany). Cell viability was assessed by comparing the absorbance values of the treated wells to those of the control group.

### 2.9 In Vivo Efficacy and Safety Evaluation

#### 2.9.1 Animals

Male C57BL/6 mice (10 weeks old; 25.0–30.0 g; specific pathogen-free grade) were purchased from Changsheng Biotechnology (Shenyang, China). All animal procedures were conducted in accordance with institutional and national guidelines for the care and use of laboratory animals and were approved by the Institutional Animal Care and Use Committee of Shenyang Pharmaceutical University (SYPU-IACUC-S2025-1121-102). Animals were housed in a controlled environment (22 ± 1 °C, 50 ± 2% relative humidity, 12 h light/dark cycle) and were maintained on a standard pelleted diet and *ad libitum* access to drinking water.

#### 2.9.2 Drug Administration and Treatments

After a 7-day acclimatization period, the mice were randomly assigned into six groups (*n* = 8 per group): Group 1 (G1): Normal group receiving only phosphate-buffered saline (PBS, pH 7.4); Group 2 (G2): TNF-α group receiving PBS + TNF-α; Group 3 (G3): Free Ole group (20 mg/kg/day Ole + TNF-α); Group 4 (G4): Low-dose CS-Ole-Lip group (2 mg/kg/day CS-Ole-Lip + TNF-α); Group 5 (G5): High-dose CS-Ole-Lip group (20 mg/kg/day CS-Ole-Lip + TNF-α); and Group 6 (G6): Ole-Lip group (20 mg/kg/day Ole-Lip + TNF-α).

Assigned interventions were administered once daily via oral gavage for 10 consecutive days (days 1–10). To assess preventive efficacy, acute vascular inflammation was induced by intraperitoneal TNF-α administration once daily (25 μg/kg/day) from day 4 through day 10 in G2–G6, whereas G1 received the same volume of PBS (pH 7.4). Body weight was recorded daily throughout the study. Two hours after the final TNF-α injection on day 10, mice were euthanized and whole blood was collected for serum preparation. Serum was obtained by centrifugation and stored at −80 °C until subsequent analysis. Of the 8 mice per group, serum samples from 5 mice were used for MCP-1 and IL-6 analyses. Serum samples from the remaining 3 mice were used for alanine aminotransferase (ALT), aspartate aminotransferase (AST), blood urea nitrogen (BUN), and creatinine (CREA) analyses, and the same 3 mice were used for histopathological evaluation. All designated samples were included in the respective analyses, with no exclusions.

#### 2.9.3 Expression of Proinflammatory Cytokines in Serum

Whole blood was allowed to clot at RT (25.0 ± 2.0 °C), and serum was collected by centrifugation at 3000 rpm for 5 min at 4 °C. Serum concentrations of MCP-1 and IL-6 were quantified using commercially available ELISA kits (Invitrogen, Thermo Fisher Scientific, Waltham, MA, USA) according to the manufacturer’s instructions. Analyte concentrations were determined by interpolation from assay-specific standard curves. Serum MCP-1 and IL-6 concentrations were analyzed using five mice per group (*n* = 5).

#### 2.9.4 Safety Evaluation

Serum samples were collected as described in Section 2.9.3. Serum biochemical markers of hepatic and renal function, including ALT, AST, BUN, and CREA, were quantified. Serum biochemical markers were evaluated using three mice per group (*n* = 3), with no exclusions. Histopathological evaluation was performed on the same three mice, and representative sections are presented for G1, G3, and G5, selected to compare the normal control, the free-drug treatment, and the highest-dose CS-Ole-Lip treatment. Major organs (heart, liver, spleen, lungs, and kidneys) were subsequently harvested and processed for histopathological examination using hematoxylin and eosin (H&E) staining. The main biochemical comparison focused on G1 and the treated groups (G3−G6), because this assay was designed to evaluate potential hepatic and renal toxicity associated with the oleuropein formulations. To provide context for the effect of TNF-α challenge alone, biochemical marker values for G2 are reported separately in Supporting Information Table S3.

### 2.10 Statistical Analysis

Data are presented as mean ± SD for nanoparticle physicochemical characterization and in vitro experiments, and as mean ± SEM for in vivo studies unless otherwise stated. Group differences were assessed by one-way ANOVA followed by the appropriate post hoc comparisons. For Figure 7, each formulation series was analyzed independently using one-way ANOVA followed by Dunnett’s multiple-comparisons test against the corresponding untreated (zero-concentration) control; no comparisons were statistically significant (*p* > 0.05). For Figure 8, Bonferroni-corrected pairwise comparisons were performed specifically for G1 versus G2 and for G2 versus each treatment group (G3–G6). Statistical analyses and graphical visualization were performed using GraphPad Prism 9.4 (GraphPad Software, San Diego, CA, USA) and Origin (OriginLab, Northampton, MA, USA). A two-sided *p* value < 0.05 was considered statistically significant.

## 3 Results and Discussion

### 3.1 Response Surface Model via BBD

Formulation optimization was performed first to define a CS-Ole-Lip system with controlled Ole content and reproducible baseline colloidal attributes, which is required to interpret TNF-α-responsive anti-inflammatory outcomes without confounding from dose variability. BBD was selected to evaluate three formulation variables that provide orthogonal control over composition and surface structure: Ole amount (*A*), lecithin-to-cholesterol mass ratio (*B*), and CS concentration (*C*). Factor *A* defines the drug level introduced during preparation and sets the payload range explored, Factor *B* controls membrane composition through cholesterol-dependent bilayer packing, and Factor *C* specifies the polymer available for coating formation and thus the extent of surface modification. Responses were chosen to establish a quantitative baseline before retention and bioactivity studies. Particle size (*Y*_1_) and PDI (*Y*_2_), measured in ultrapure water under standardized conditions, were used to quantify the initial dispersion state and population uniformity at preparation. Loading capacity (LC, *Y*_3_) quantified Ole incorporated per carrier mass, providing a defined starting payload against which subsequent retention and efficacy outcomes can be interpreted. Across the BBD space (Table 2), *Y*_1_ ranged from 159 to 303 nm, *Y*_2_ from 0.19 to 0.33, and *Y*_3_ from 4.5% to 13.7%, indicating that the selected factor ranges generated substantial separation in both dispersion attributes and Ole payload within the experimental domain.

**Table 2:** BBD Matrix with the Actual Response Values for the Optimization of CS-Ole-Lip^a^

	Factors			Responses		
Std order	*A*	*B*	*C*	*Y*_1_	*Y*_2_	*Y*_3_
1	10	2	0.075	275 ± 11	0.27 ± 0.03	6.79 ± 2.40
2	20	2	0.075	258 ± 33	0.33 ± 0.08	11.18 ± 2.78
3	10	5	0.075	159 ± 14	0.21 ± 0.01	5.68 ± 1.90
4	20	5	0.075	184 ± 21	0.23 ± 0.03	13.24 ± 0.90
5	10	3.5	0.05	160 ± 14	0.19 ± 0.03	4.94 ± 0.80
6	20	3.5	0.05	171 ± 12	0.19 ± 0.01	13.69 ± 2.33
7	10	3.5	0.1	184 ± 11	0.22 ± 0.01	4.50 ± 0.59
8	20	3.5	0.1	186 ± 14	0.24 ± 0.02	9.36 ± 2.28
9	15	2	0.05	235 ± 11	0.30 ± 0.06	7.51 ± 1.04
10	15	5	0.05	162 ± 5	0.20 ± 0.04	8.45 ± 0.53
11	15	2	0.1	303 ± 15	0.31 ± 0.06	9.12 ± 0.23
12	15	5	0.1	185 ± 16	0.25 ± 0.07	9.02 ± 1.25
13^b^	15	3.5	0.075	178 ± 20	0.21 ± 0.03	7.93 ± 1.85
14^b^	15	3.5	0.075	167 ± 12	0.20 ± 0.01	8.64 ± 0.48
15^b^	15	3.5	0.075	169 ± 13	0.20 ± 0.01	9.05 ± 0.76

a Abbreviations: *A* = Amount of Ole (mg); *B* = Lecithin/cholesterol mass ratio; *C* = CS concentration (mg/mL); *Y*_1_: Particle size (nm), *Y*_2_: PDI, and *Y*_3_: LC (%).

b Replicated center points of the BBD.

The BBD data set was then fitted to polynomial models to quantify factor effects and support numerical optimization. Model adequacy was evaluated by ANOVA using prespecified criteria: significant regression (*p* < 0.05), nonsignificant lack-of-fit (*p* > 0.05), agreement between adjusted and predicted *R^2^* (difference ≤ 0.2), and adequate precision >4.^28^ All selected response surface methodology (RSM) models satisfied these requirements (Table S1). With model validity established, response surface analysis was used to visualize how *A*, *B*, and *C* jointly influence *Y*_1_, *Y*_2_, and *Y*_3_. The 3D response surface plots (Figure 2) summarize the main effects and interaction patterns that define the trade-offs between dispersion uniformity and Ole incorporation, providing the quantitative basis for identifying an operating region appropriate for downstream retention studies and TNF-α-responsive efficacy assessment.

**Figure 2:**
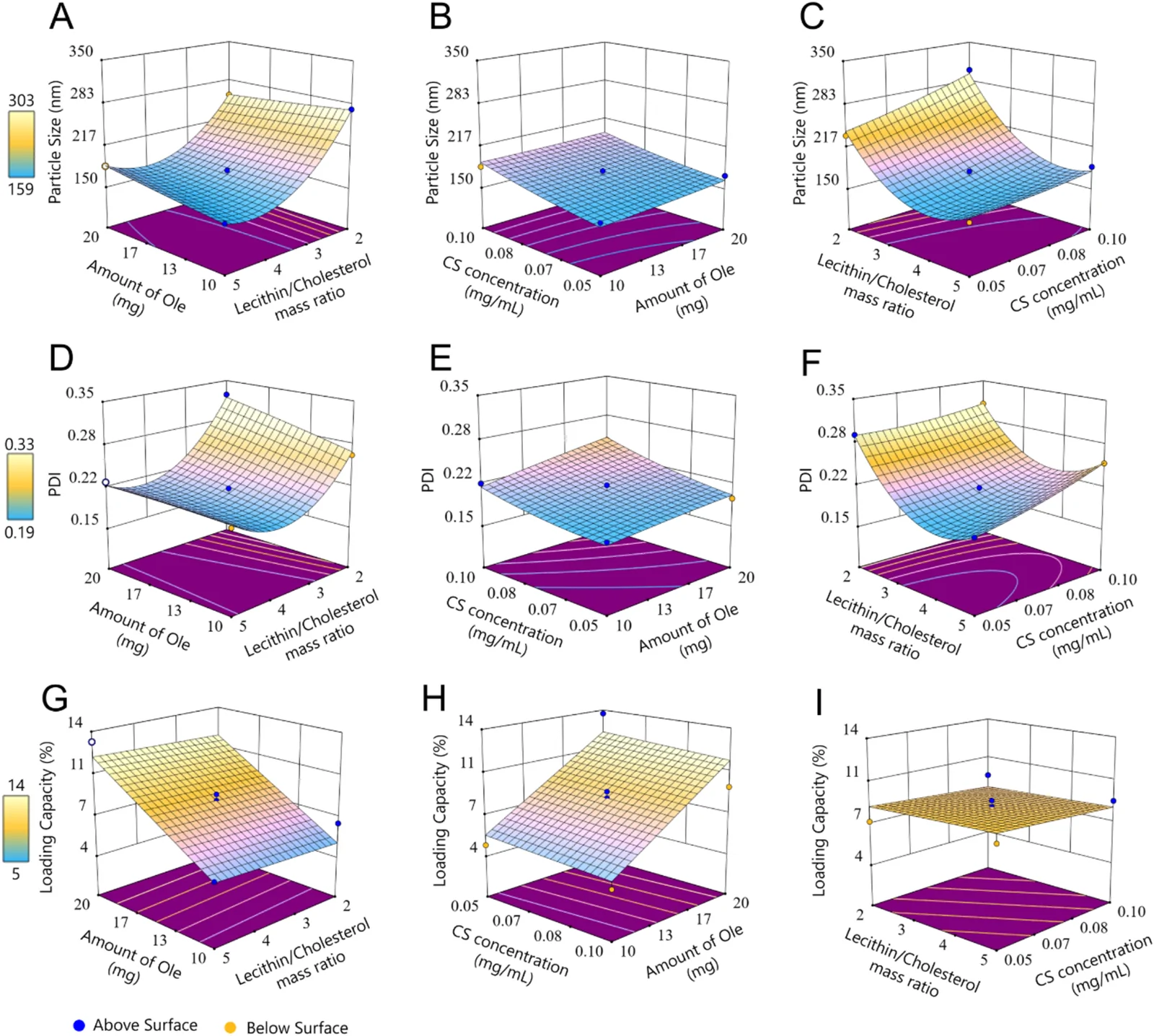
Response surface plots illustrating the effects of the amount of Ole, lecithin/cholesterol mass ratio, and CS concentration on (A–C) particle size (*Y*_1_), (D–F) PDI (*Y*_2_), and (G–I) LC (*Y*_3_).

In evaluating interaction effects of independent variables on response variables, *Y*_1_ was adequately captured by a second-order RSM model, indicating that particle size in the CS-Ole-Lip system is governed by coupled compositional and interfacial assembly processes rather than by independent, monotonic factor effects (Figure 2A–C; eq 5). *B* produced the largest magnitude linear effect on *Y*_1_ (−47.65), establishing the lecithin-to-cholesterol mass ratio as the dominant size-controlling factor within the explored space. This dependence is chemically consistent with sterol-dependent modulation of bilayer packing and membrane physical properties, where changes in cholesterol content can alter lipid chain ordering and curvature-sensitive membrane behavior, thereby influencing vesicle assembly and the resulting hydrodynamic size distribution.^29,30^ The strong positive curvature (+46.60) indicates a pronounced nonlinear response, supporting the presence of a composition window that favors smaller particle sizes and flanking regions where size increases as membrane rigidity and restructuring behavior change. *C* increased *Y*_1_ through a positive main effect (+16.28) with mild convexity (+3.04), consistent with CS coating increasing hydrodynamic diameter through growth of the interfacial layer and, at higher polymer availability, greater propensity for polymer-mediated association.^31^ In contrast, *A* contributed only a small positive effect (+2.71) with weak curvature (+0.8938), indicating that the amount of Ole has limited direct influence on size under average formulation conditions and primarily modulates size through composition-dependent interfacial effects rather than as an intrinsic driver of vesicle enlargement.

5 $$Y_{1}=171.51+2.71A-47.65B+16.28C+0.8938A^{2}+46.60B^{2}+3.04C^{2}+10.60\mathrm{AB}-2.04\mathrm{AC}-11.10\mathrm{BC}$$

The dominant interaction was *BC* (−11.10), indicating that the size consequence of CS depends strongly on bilayer composition. This observed behavior is supported by the established role of cholesterol in modulating bilayer packing, ordering, rigidity, and liposome stability, thereby altering the interfacial conditions that govern CS adsorption and particle association during coating.^32–34^ The positive *AB* interaction (+10.60) indicates that the size impact of Ole input is amplified as *B* increases, which could be from composition-dependent partitioning and packing effects that change how Ole perturbs interfacial organization.^35^ The *AC* interaction was smaller and negative (−2.04), supporting comparatively weak coupling between Ole input and CS concentration relative to the stronger, composition-mediated coating effects captured by *BC*. In parallel with particle size, *Y*_2,_ also known as the dispersion homogeneity response, was best described by a second-order RSM model, showing that *Y*_2_ arises from nonlinear interactions among formulation composition and CS coating conditions during nanoliposome formation (Figure 2D–F; eq 6).

6 $$Y_{2}=0.2057+0.0130A-0.0388B+0.0190C-0.0012A^{2}-0.0531B^{2}+0.0053C^{2}-0.0120\mathrm{AB}+0.0048\mathrm{AC}+0.0105\mathrm{BC}$$

*B* exerted the largest main effect on *Y*_2_, with a negative linear coefficient (−0.0388) and a stronger negative quadratic term (−0.0531), indicating that the lecithin-to-cholesterol ratio is the dominant determinant of dispersion uniformity within the explored design space. The additional magnitude of the *B* ^2^ term relative to *A* ^2^ and *C* ^2^ indicates that PDI is particularly sensitive to composition-dependent nonlinearity, suggesting that relatively small shifts in *B* can markedly alter the breadth of the size distribution. This pattern is similarly rationalized by the role of membrane composition in regulating bilayer packing and mechanical stability during hydration and dispersion, where changes in membrane composition can shift the system from relatively uniform vesiculation to broader, multipopulation formation. *C* exhibited a positive main effect on *Y*_2_ (+0.0190), consistent with increasing CS availability increasing heterogeneity, potentially by introducing variation in coating thickness or promoting transient polymer-mediated association. The positive curvature for *C* ^2^ (+0.0053) suggests mild convex behavior, indicating that these effects may intensify as CS concentration increases. *A* also contributed a positive main effect (+0.0130), indicating that higher Ole modestly increases polydispersity, whereas the *A* ^2^ term was negligible in magnitude (−0.0012), supporting limited curvature in the drug input dimension and implying that drug feed affects uniformity primarily through first-order changes.

The *AB* interaction was negative (−0.0120), which suggests that increasing *A* could partially offset the *Y*_2_ -reducing tendency associated with higher *B*, or conversely that the *Y*_2_ penalty from higher *A* is more apparent when *B* is low. The *BC* interaction was positive (+0.0105), indicating that the CS-associated increase in *Y*_2_ becomes more pronounced as *B* increases, which could reflect composition-dependent shifts in polymer adsorption and interfacial organization that broaden coating heterogeneity. The *AC* interaction was smaller and positive (+0.0048), consistent with comparatively weak coupling between the amount of Ole and CS concentration, with any joint contribution to dispersion breadth likely secondary to the stronger composition-driven effects associated with *B* and its interactions.

In contrast to the colloidal responses, *Y*_3_ followed a first-order RSM model, indicating that payload incorporation in the CS-Lip matrix is dominated by direct formulation composition effects within the studied range, with no substantial nonlinear or interaction-driven behavior detected (Figure 2G–I; eq 7).

7 $$Y_{3}=8.60+3.20A+0.2125B-0.3125C$$

A produced the dominant positive effect on *Y*_3_ (+3.20), establishing *A* as the primary determinant of % loading capacity. This interpretation is mechanistically plausible because, under fixed process conditions, drug availability governs the amount incorporated per unit carrier mass. Across the studied domain, the response indicates a drug availability-limited regime in which increasing feed increased incorporation, with no saturation behavior detectable at the model resolution. *B* contributed a smaller positive effect on *Y*_3_ (+0.2125), indicating that lecithin-to-cholesterol ratio exerts a modest influence on loading. This dependence accords with membrane composition subtly modulating the partitioning environment available for Ole during liposome formation, where lipid packing and bilayer organization can affect the capacity for association or retention of drug during hydration and subsequent handling. In contrast, *C* showed a small negative effect (−0.3125), suggesting that higher CS concentration slightly reduces loading capacity. This behavior may arise due to the condition-dependent nature of CS-Lip interactions, which are influenced by membrane composition/phase state and interfacial properties that govern adsorption and complexation. Prior reports showing distinct CS-Lip complexation outcomes under different membrane states further support the plausibility of a composition-dependent CS effect on loading capacity in the present system.^36^

The mechanistic contrast among the three response models is itself informative about the behavior of this CS-Ole-Lip system and its implications for future formulation development. *Y*_1_ and *Y*_2_ both required second-order polynomial models, reflecting the nonlinear coupled effects of membrane composition and coating concentration on interfacial vesicle assembly, processes in which relatively small compositional shifts can produce nonadditive changes in particle size distribution and dispersion uniformity. In contrast, *Y*_3_ followed a simple first-order linear model, establishing that within the studied range, Ole incorporation is governed primarily by drug feed availability rather than by the coupled interfacial assembly dynamics that control the colloidal responses. This distinct mechanistic regime has practical implications: future modifications to lecithin/cholesterol ratio or CS concentration intended to tune particle size will have limited, predictable effects on Ole payload, which can instead be controlled independently through the Ole input quantity during preparation. The practical significance of the three optimized response values connects the formulation properties with the downstream biological studies that follow. Specifically, *Y*_1_ (168.73 nm) places the formulation within the sub-200 nm range associated with enhanced mucosal adhesion, intestinal epithelial transcytosis, and endothelial cell internalization, all relevant to the intended oral-to-vascular delivery route. *Y*_2_ (PDI = 0.17) indicates a monomodal population essential for reproducible release kinetics and consistent dose delivery across individual animals in the in vivo model. Finally, *Y*_3_ (LC = 12.22%) establishes the Ole payload per unit carrier mass that determines the Ole concentrations in the dosing suspensions to be used in subsequent experiments. Preliminary range-finding trials conducted prior to the Box-Behnken design indicated that Ole amounts above 20 mg (25–40 mg) resulted in visible precipitation of the liposomal suspension, and CS concentrations below 0.05 mg/mL (0.025 and 0.0125 mg/mL) resulted in unstable, highly variable zeta potential values; these observations informed the factor ranges selected for the design space (Table 1).

### 3.2 Optimization and Validation

The above interaction analysis shows that response behavior is governed by coupled changes in membrane composition and surface modification, producing nonadditive shifts in size distribution and payload. This motivates a model-guided optimization step to identify a single operating point that satisfies the multiresponse criteria simultaneously. The optimized condition and its confirmatory outcomes are summarized in Table 3. The optimized settings, 20 mg Ole amount, a lecithin-to-cholesterol mass ratio of 3.97:1, and 0.05 mg/mL CS, define a composition expected to control dispersion state while maintaining adequate payload incorporation. Numerical optimization identified this compromise solution with an overall desirability of D = 0.899. The predicted LC at this compromise point (12.2%) is lower than the highest single-run LC in the design matrix (13.69%, run 6, Table 2), because the desirability function constrained particle size and PDI simultaneously rather than maximizing LC alone. Model reliability at this point was evaluated using the 95% prediction intervals and by preparing the formulation in six independent replicates to capture run-to-run variability.

**Table 3:** Optimal Conditions and Analysis of the Predicted and Observed Values^a^

Factors	Optimized levels
*A*: Amount of Ole (mg)	20
*B*: Lecithin/Cholesterol Mass Ratio	3.97:1
*C*: CS Concentration (mg/mL)	0.05

Responses	Predicted value	95% PI low	Observed value	95% PI high	% Error
*Y*_1_ = Particle size (nm)	160.4	139.6	168.73 ± 3.86	181.9	5.19
*Y*_2_ = PDI	0.18	0.16	0.17 ± 0.01	0.20	5.56
*Y*_3_ = LC (%)	12.2	10.7	12.22 ± 0.25		

a Notes: Overall desirability at the optimum, *D* = 0.899. 95% PI = 95% prediction interval generated by Design-Expert for a single future measurement; observed values are mean ± SD of six confirmatory replicates (*n* = 6).

All observed means fell within their respective 95% prediction intervals, with errors below 10% across responses. *Y*_1_ showed close agreement with the model (5.19% error), supporting accurate capture of the dominant determinants of hydrodynamic size at the optimum. *Y*_2_ was similarly well predicted (5.56% error), indicating that the optimized setting yields a reproducible, monomodal dispersion. *Y*_3_ exhibited near-identical agreement between prediction and experiment (0.16% error), consistent with well-resolved parametrization of Ole incorporation at the optimized composition and low sensitivity to run-to-run variation.

### 3.3 Structural and Physicochemical Characterization of the Optimized Formulation

Following numerical optimization and experimental confirmation, the optimized CS-Ole-Lip formulation was subjected to structural and physicochemical characterization to verify key material attributes and define parameters relevant to dispersion stability and payload performance. The optimized system showed a hydrodynamic diameter of 168.73 ± 3.86 nm (Figure 3A) with a PDI of 0.17 ± 0.01, indicating a relatively narrow size distribution. The zeta potential was −22.50 ± 2.39 mV (Figure 3B), consistent with a moderately negative surface charge.

**Figure 3:**
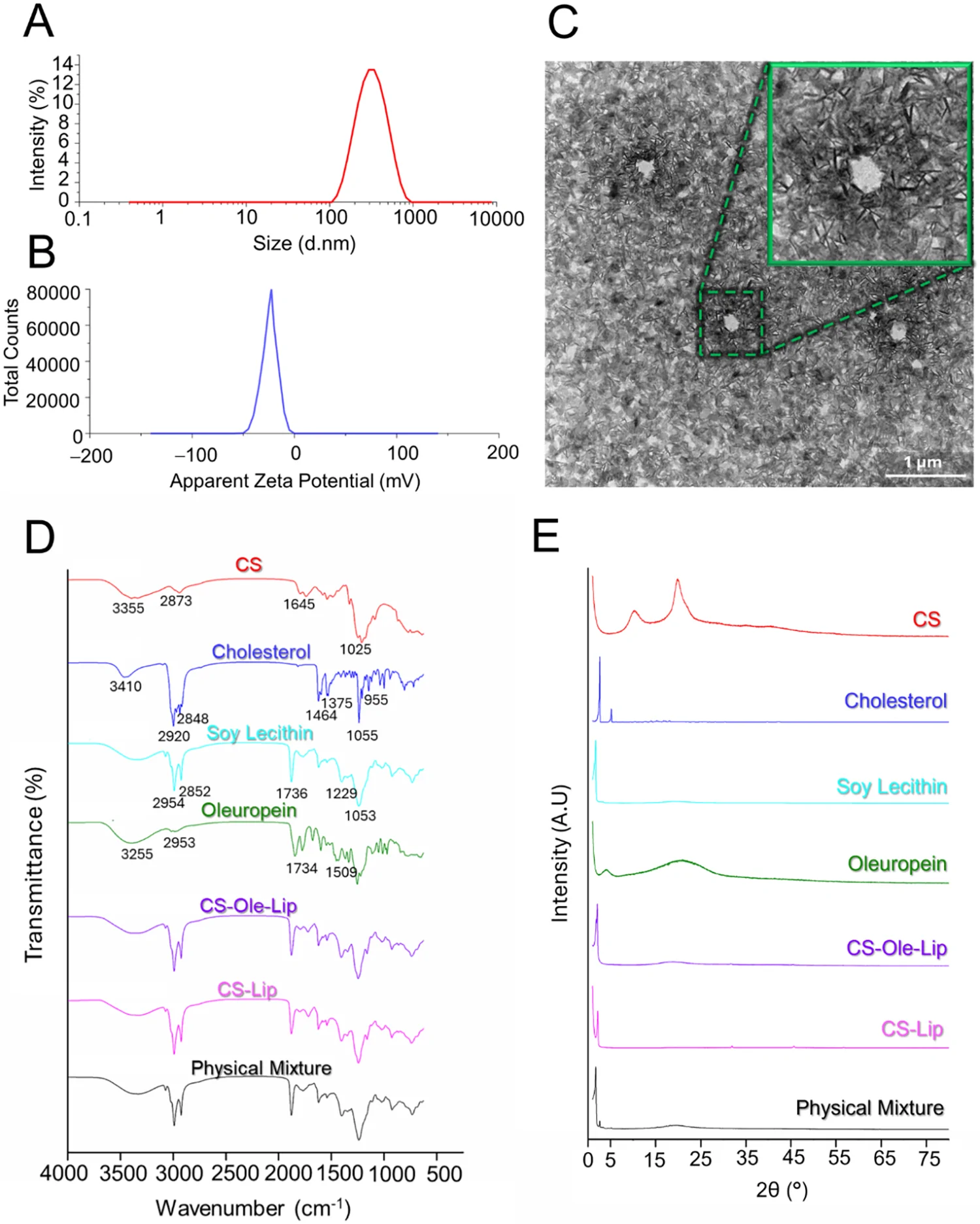
Physicochemical characteristics of CS-Ole-Lip: (A) particle size distribution, (B) zeta potential distribution, (C) TEM micrograph (×11,000; scale bar = 1 μm), (D) FTIR and (E) XRD profiles of CS-Ole-Lip, physical mixture, and individual components.

Although CS is cationic, the negative zeta potential is mechanistically plausible and may be explained in two ways: first, protonation of CS amino groups is pH-dependent, and partial deprotonation at neutral to slightly basic conditions reduces the positive surface contribution.^14^ Second, negatively charged lipid components in the formulation, including phospholipid headgroups, can dominate the net interfacial charge and outweigh the contribution of CS, resulting in an overall negative zeta potential.^16,37^ As a direct control for CS coating, the zeta potential of uncoated Ole-Lip was measured as −24.65 ± 1.09 mV (*n* = 6), compared with −22.50 ± 2.39 mV (*n* = 6) for CS-Ole-Lip. The shift toward a less negative value upon CS addition is directionally consistent with partial surface charge neutralization by adsorbed CS, but the difference did not reach statistical significance (unpaired two-tailed *t* test, *p* = 0.07). We therefore do not present the zeta potential shift as independent evidence of coating and instead rely on the FTIR and XRD data described below as the primary indication of CS association with the liposome surface. In addition, we note that this net negative zeta potential reflects the balance of surface charge integrated over the entire particle and does not preclude a fraction of CS amino groups remaining protonated at the polymer–lipid interface; this local, partially protonated character underlies the short-range electrostatic interactions described in Sections 3.5 and 3.6. At this magnitude, the zeta potential suggests sufficient electrostatic repulsion to support colloidal stability and reduce aggregation tendency during storage and handling, which is important for preserving nanoparticle performance.^37^ In terms of payload metrics, the optimized formulation achieved an EE of 87.17 ± 0.27% and a LC of 12.22 ± 0.25%, indicating efficient Ole incorporation under the optimized preparation conditions.

TEM imaging provided qualitative morphological evidence of nanoscale vesicular particles with generally spherical morphology (Figure 3C). The needle-like textured background observed in Figure 3C is most consistent with crystallization of PBS salts during negative staining, as the sample was imaged directly from the PBS-based dispersion without prior dialysis or buffer exchange; this preparation artifact limits morphological interpretation, and the retained image cannot exclude sample heterogeneity. The apparent particle size observed by TEM was smaller than the hydrodynamic diameter measured by dynamic light scattering (DLS), which is expected because DLS measures hydrated particles in suspension, including the solvation layer and any surface-associated polymer contribution, whereas TEM visualizes dried particles under vacuum conditions after staining.^38^

FTIR analysis further substantiated assembly of the CS-Ole-Lip system at the molecular level through band broadening, shifts, and intensity attenuation relative to the individual components (Figure 3D). In particular, the broad band near 3355 cm^–1^, assigned to overlapping O–H and N–H stretching vibrations of CS, became broader and slightly shifted in the nanoparticle spectrum, which is consistent with altered hydrogen bonding interactions after formulation, suggesting that CS hydroxyl and amino groups participated in intermolecular interactions within the assembled carrier, rather than behaving as free/unassociated CS. Moreover, the Ole-associated C=O band at 1734 cm^–1^ decreased in intensity in the formulated system, while the CS amide I band around 1645 cm^–1^ was also attenuated. Although FTIR alone does not define the exact binding mode, the concurrent changes in these bands support noncovalent interactions, most plausibly hydrogen bonding and secondary polar interactions, among Ole, CS, and the lipid phase. Importantly, the spectrum of the CS-Ole-Lip showed band overlap and reduced signal definition relative to the isolated raw materials, which argues against simple spectral superposition of physically separated constituents. Instead, these spectral features support successful incorporation of Ole within an interacting polymer–lipid matrix and are consistent with complexation in the optimized formulation. XRD analysis (Figure 3E) further supports this observation wherein characteristic sharp crystalline peaks of Ole, CS, soy lecithin, and cholesterol were evidently reduced in the diffractogram of CS-Ole-Lip. Instead, a broad diffuse peak around 20° (2θ) was observed, indicative of an amorphous structure. This transition from crystalline to amorphous form confirms that Ole was incorporated within the lipid–polymer matrix in a less crystalline state, which is consistent with successful nanoparticle formation.

### 3.4 Colloidal Stability

The colloidal stability of nanoparticulate drug carriers is a critical factor influencing their shelf life, delivery performance, and clinical applicability. In this study, the stability of the optimized CS-Ole-Lip was systematically assessed under refrigerated (4.0 ± 2.0 °C) and RT (25.0 ± 2.0 °C) conditions over a three-month period. The parameters evaluated included particle size, PDI, zeta potential, and EE, all of which serve as indicators of nanoparticle integrity and storage stability.

As shown in Figure 4A–C, CS-Ole-Lip maintained relatively consistent particle size, PDI, and surface charge under refrigeration, with minimal fluctuations observed throughout the 3-month storage period. This suggests a stable colloidal system with low aggregation propensity at reduced temperatures.^16^ In contrast, samples stored at 25 °C showed a statistically significant increase in particle size and PDI as early as one month of storage (*p* < 0.05), suggesting the onset of structural destabilization and particle aggregation. This temperature-dependent behavior likely reflects the impact of increased molecular motion and collision frequency at higher thermal energy, which promotes vesicle fusion or swelling—a phenomenon commonly reported in polymer-coated nanocarriers.^36,39,40^

**Figure 4:**
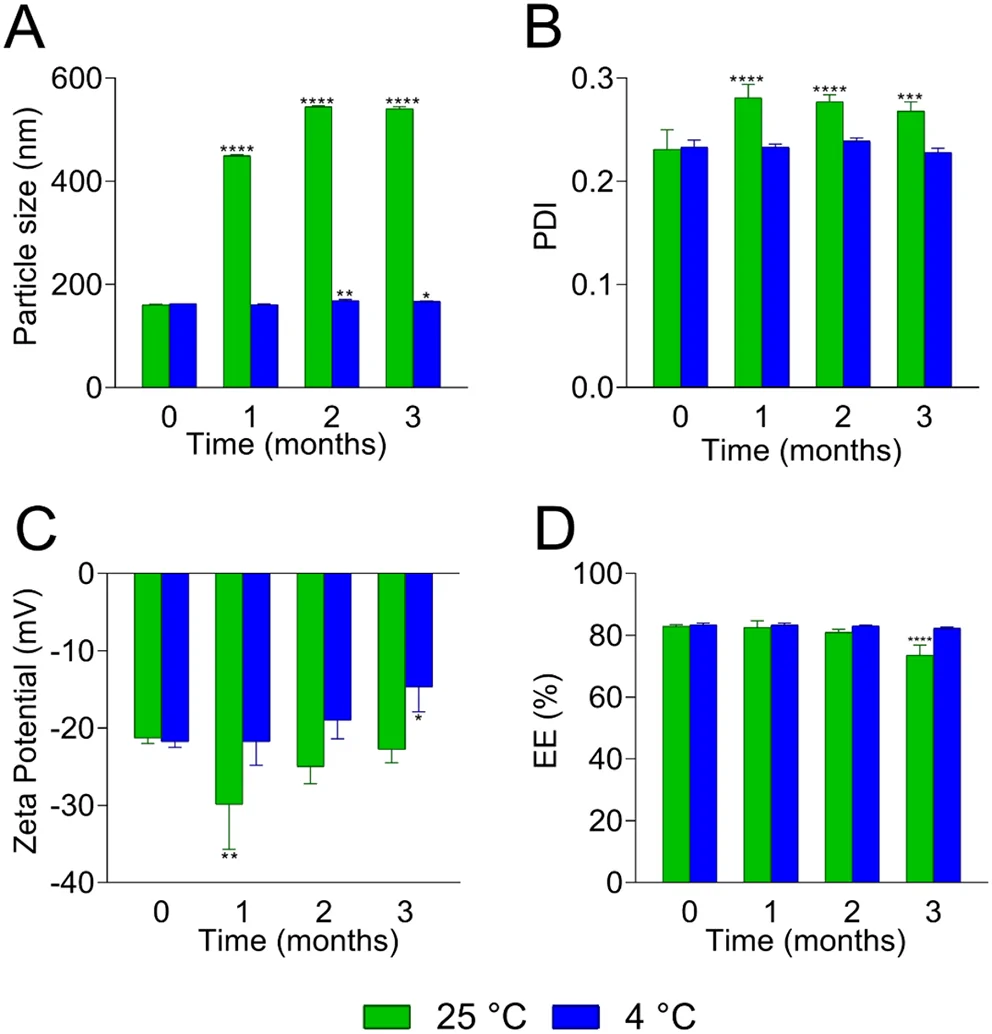
Colloidal stability of CS-Ole-Lip stored at 4.0 ± 2.0 °C and 25.0 ± 2.0 °C for 3 months. (A) Particle size, (B) PDI, (C) zeta potential, and (D) encapsulation efficiency (EE) were measured at defined intervals. Data are presented as mean ± SD (*n* = 3). *p < 0.05, **p < 0.01, ***p < 0.001, and ****p < 0.0001 compared to the initial formulation characteristics on day 0.

Similarly, EE values remained largely unchanged at 4.0 ± 2.0 °C, suggesting effective retention of Ole within the lipid bilayer under cold storage. However, a noticeable decline in EE was observed at 25.0 ± 2.0 °C, particularly during the later stages of storage. This reduction may be attributed to progressive Ole leakage from the liposomal matrix, potentially accelerated by lipid rearrangement or CS loosening at elevated temperatures.^41,42^

These findings reinforce the importance of low-temperature storage in preserving the physicochemical attributes of CS-Ole-Lip. The results demonstrate that refrigerated storage is preferable over the three-month period evaluated for minimizing particle destabilization and active compound loss, particularly for temperature-sensitive polyphenolic drugs like Ole. These findings are consistent with previous reports where nanoparticle destabilization was driven by temperature-dependent changes in hydrophilic–hydrophobic interactions and surface charge dynamics.^43,44^ It should be noted that the present stability assessment monitors colloidal attributes (particle size, PDI, zeta potential) and Ole retention (EE) but does not directly quantify Ole chemical degradation products. The observed decline in EE at 25 °C could reflect either physical leakage of intact Ole from the liposomal matrix or chemical degradation of Ole within the carrier.

### 3.5 In Vitro Release Study

To evaluate the release characteristics of Ole from the CS-Ole-Lip, an in vitro release study was conducted in SGF (pH 1.2), SIF (pH 6.8), and physiological buffer (pH 7.4) to compare how the formulation behaves as pH shifts from the stomach to the intestine and then to physiological conditions (Figure 5). The study was extended to 96 h to capture the full sustained release phase and approach to a plateau, rather than to replicate gastrointestinal transit time. Release increased steadily in all media and followed a biphasic pattern, with an early rise followed by a slower phase approaching a plateau. Cumulative release increased from ∼2–3% at 0.5 h to ∼20–23% at 6 h, and no differences were detected within this early window (*p* > 0.05). At 12 h, values remained closely aligned at 37.7 ± 0.8% (pH 1.2), 35.4 ± 1.3% (pH 6.8), and 39.7 ± 1.9% (pH 7.4) (*n* = 3), consistent with early release dominated by an accessible Ole fraction. Clear pH effects appeared later, when transport is dominated by diffusion through the liposomal membrane and the CS coating. From 8–12 h, release at pH 7.4 became higher than at pH 6.8 (*p* < 0.05), and by 48–96 h all media were distinct (*p* < 0.0001). At 96 h, cumulative release reached 55.0 ± 1.3% at pH 7.4, 48.5 ± 2.2% at pH 1.2, and 43.6 ± 1.6% at pH 6.8, giving the late-phase order pH 7.4 > pH 1.2 > pH 6.8. Practically, this means the formulation limits rapid early leakage, then releases Ole gradually in a pH-dependent manner as exposure continues.

**Figure 5:**
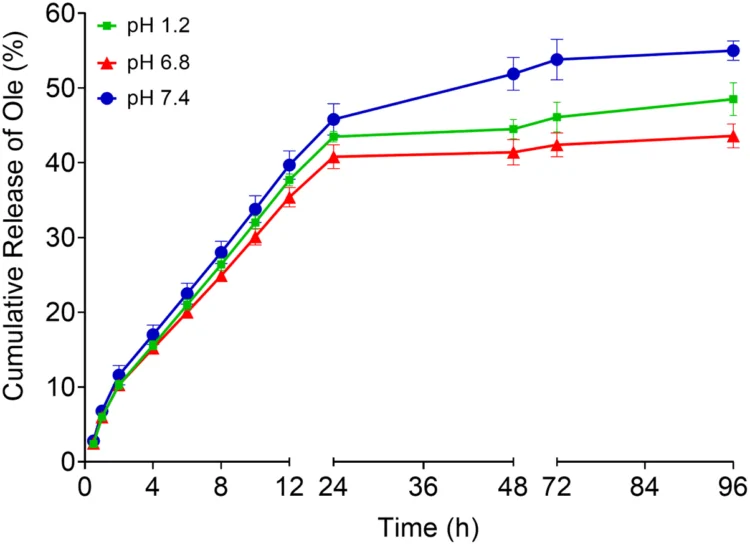
In vitro cumulative release profiles of CS-Ole-Lip in simulated gastric fluid (pH 1.2), intestinal fluid (pH 6.8), and physiological buffer (pH 7.4) at 37 °C over time. Data are presented as mean ± SD (*n* = 3).

The 55.0 ± 1.3% cumulative release at pH 7.4 after 96 h indicates a sustained and incomplete release profile for CS-Ole-Lip. Although previous studies on chitosan- or polymer-coated phospholipid vesicles commonly evaluated release over shorter periods, their findings support the mechanistic interpretation observed in the present study. For instance, CS-coated genistein-loaded liposomes showed reduced drug release under acidic gastric conditions and controlled release under intestinal pH, with release behavior attributed to Fickian diffusion.^17^ Similarly, multilayered quercetin-loaded liposomes demonstrated that increasing polymer coating layers reduced drug release, indicating that the external polymer layer can act as a diffusion-controlling barrier.^45^ In the present formulation, the low early phase release (∼20–23% at 6 h), followed by gradual progression toward a plateau, suggests that Ole release was restricted by both its retention within the phospholipid bilayer and the barrier effect of the chitosan coating. This sustained, incomplete release behavior may be relevant for the intended oral delivery application, as it may limit the risk of premature drug release during gastric exposure while maintaining sustained Ole availability under intestinal pH conditions. However, whether the released Ole fraction translates to pharmacologically sufficient systemic exposure depends on absorptive processes at the intestinal epithelium, which are beyond the scope of the in vitro dialysis model and will be assessed directly in the planned pharmacokinetic study.

This ordering is compatible with a CS-Lip because the coating is an interfacial layer whose barrier strength depends on CS ionization and how tightly it remains associated with the liposome surface. At pH 6.8, CS is still partly protonated and can remain strongly associated with negatively charged lipid headgroups, forming a more coherent barrier that slows diffusion.^46,47^ At pH 7.4, CS is less protonated, which can weaken interfacial cohesion and make the coating less continuous, increasing bilayer accessibility and net diffusion over time.^46,47^ The intermediate behavior at pH 1.2 is consistent with strong protonation and hydration of CS, which supports coating association but can also increase water penetration, producing neither the slowest nor the fastest release.^46–48^ Our results indicate that CS-Ole-Lip limits early Ole release in SGF and exhibits sustained, pH-dependent release behavior across the tested media.

To further elucidate the underlying release mechanism, cumulative release data were fitted to zero-order, first-order, Higuchi, Korsmeyer–Peppas, and Hixson–Crowell kinetic models using DDSolver (Table S2). The Korsmeyer–Peppas model demonstrated the best overall fit (*R* ^2^ = 0.8835–0.9227), followed by the Higuchi model (*R* ^2^ = 0.7200–0.8221), indicating a diffusion-controlled release process. The *n* values for the CS-Ole-Lip (0.3071–0.3396) remained below 0.45, confirming Fickian diffusion. This slow diffusional rate is attributed to the combined barrier effect of the lipid bilayer and the CS shell, which collectively restrict molecular transport.

These findings are consistent with previous reports on polymer-coated nanoparticle systems, where diffusion remains the dominant release mechanism when polymer erosion or matrix relaxation is minimal.^45^ In contrast to systems exhibiting anomalous transport due to extensive polymer swelling or degradation, the kinetic fits are consistent with diffusion-controlled transport; carrier integrity, however, was not measured directly during the release experiment.^14^ The poor fit of the zero-order, first-order, and Hixson–Crowell models (*R* ^2^ < 0.48) further indicates that constant-rate release and erosion-controlled mechanisms were not predominant. The observed model fit supports diffusion-dominated release under the tested in vitro conditions but does not by itself establish improved oral bioavailability. A limitation of the present release study is that the three pH media were evaluated in parallel rather than sequentially (SGF → SIF → physiological buffer). The parallel design characterizes the CS coating’s pH-dependent barrier properties at each discrete condition under controlled, decoupled conditions, but does not simulate the dynamic sequential transition in medium composition and enzyme exposure that occurs during gastrointestinal transit, a distinction that is particularly relevant for a system intended for oral administration. A sequential biorelevant cascade study using SGF with pepsin for 2 h, transferred to SIF with pancreatin for 6 h, then PBS pH 7.4 to 96 h, is planned as the next step to assess whether the CS coating maintains its release-modulating behavior under conditions more closely approximating gastrointestinal transit. Additionally, the use of enzyme-free media in the present study does not account for lipase-mediated disruption of the lipid bilayer. This enzyme effect will also be characterized in the planned sequential study.

### 3.6 In Vitro Cellular Uptake in EA.hy926 Cells

The internalization of CS-Lip was qualitatively assessed using CLSM. As shown in Figure 6, EA.hy926 endothelial cells treated with FITC-loaded CS-Lip exhibited markedly stronger green fluorescence in the cytoplasm compared to those treated with free FITC. Only the free FITC and FITC-loaded CS-Lip groups were imaged in this experiment. The left panel shows nuclear staining with Hoechst 33342 (blue), the middle panel shows FITC fluorescence (green), and the merged images illustrate intracellular localization.

**Figure 6:**
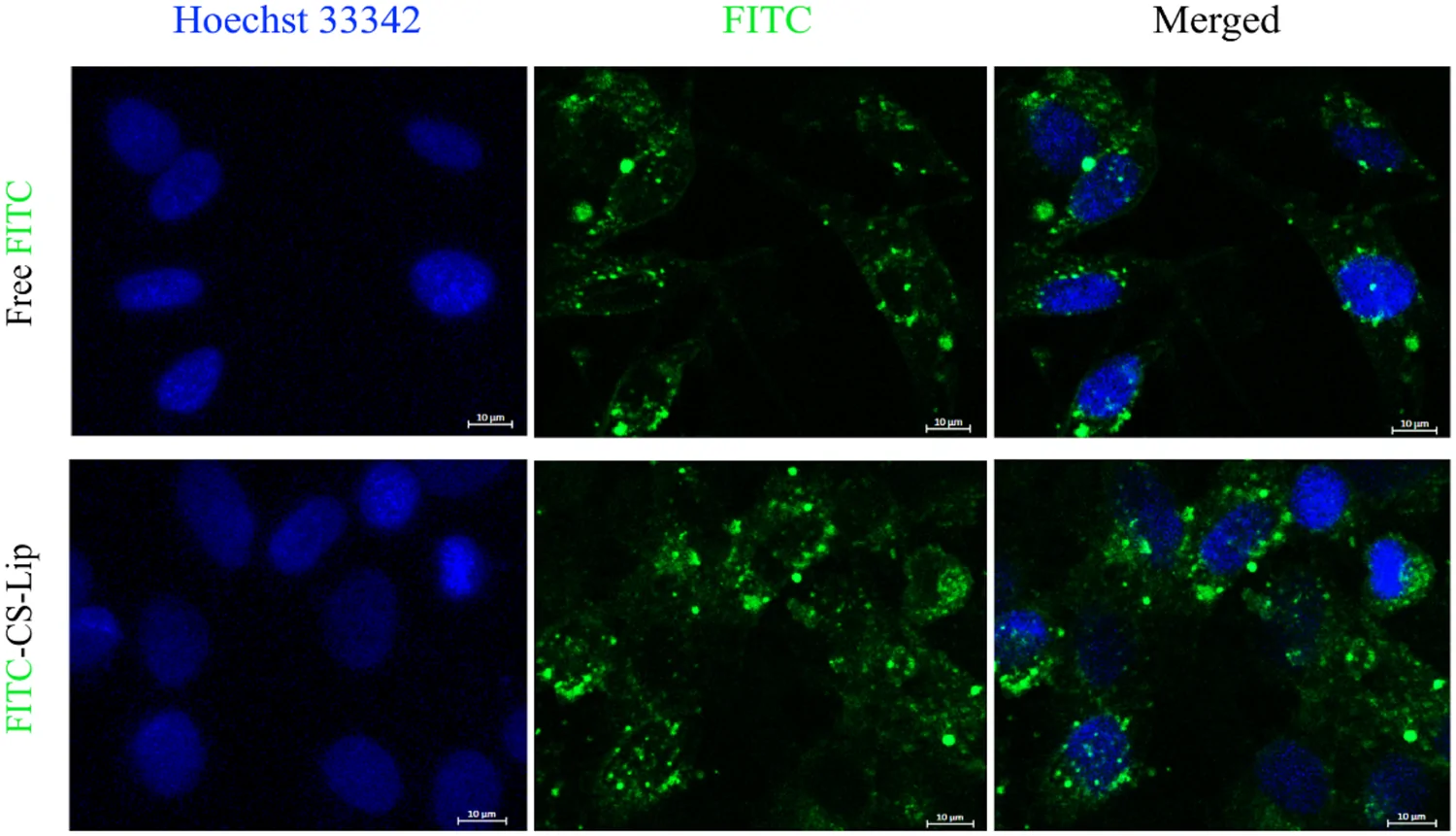
In vitro cellular uptake in EA.hy926 cells after 4 h of incubation with free FITC or FITC-loaded CS-Lip (scale bar = 10 μm). Panels show nuclear staining with Hoechst 33342 (blue), FITC fluorescence (green), and the merged image illustrating intracellular localization. FITC-loaded uncoated liposomes were not imaged in this experiment.

Confocal micrographs revealed qualitatively greater intracellular green fluorescence in EA.hy926 cells treated with FITC-loaded CS-Lip compared to those treated with free FITC, providing qualitative evidence of CS-Lip nanocarrier internalization into vascular endothelial cells. This enhanced uptake is consistent with the mucoadhesive and membrane-interactive properties of CS, in which locally protonated amino groups on the CS chain can interact electrostatically with negatively charged membrane components, facilitating adsorptive endocytosis and increasing cellular contact time.^25,46^ However, the limitations of the FITC surrogate must be acknowledged explicitly. FITC (MW 389 Da; log *P* ≈ −1.4; anionic at physiological pH) differs substantially from Ole (MW 540 Da; log *P* ≈ −0.1; neutral) in molecular weight, lipophilicity, and charge, such that its encapsulation behavior within CS-Lip, its intravesicular localization, and its intracellular redistribution following nanocarrier internalization may not replicate the behavior of Ole. Accordingly, these CLSM observations must be interpreted strictly as evidence of enhanced CS-Lip nanocarrier internalization into EA.hy926 cells, and not as a direct measure of Ole uptake efficiency or intracellular Ole distribution. Quantitative characterization via flow cytometric quantification of mean fluorescence intensity from FITC-loaded CS-Lip across treatment groups or LC-MS/MS quantification of intracellular Ole in EA.hy926 lysates may be further explored in future studies to provide rigorous cellular pharmacokinetic data for this nanocarrier system.

### 3.7 In Vitro Cytocompatibility Studies

The cytotoxicity of CS-Ole-Lip, free Ole, and blank CS-Lip was evaluated in EA.hy926 human vascular endothelial cells using the MTT assay to assess the cytocompatibility of the developed nanocarrier system. As shown in Figure 7, all formulations maintained cell viability values above 90% across the tested concentration range (500–4000 ng/mL), indicating negligible cytotoxicity.

**Figure 7:**
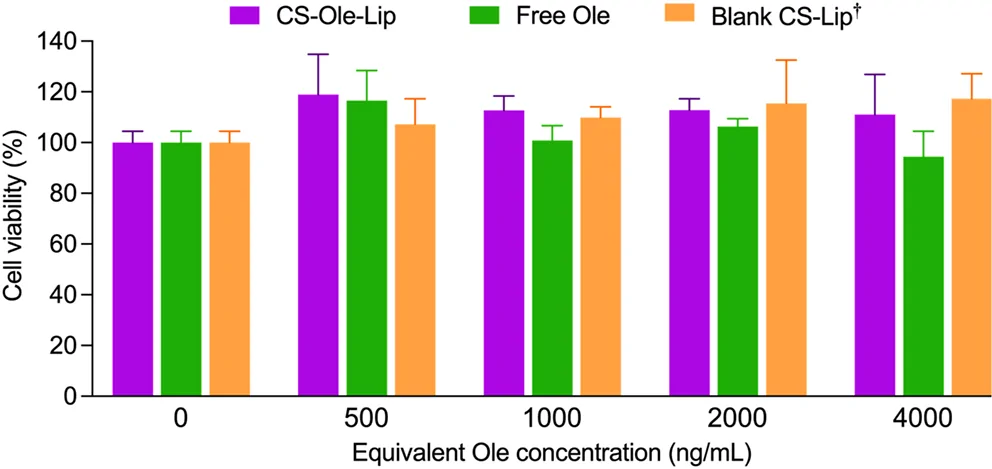
Cytotoxicity of CS-Ole-Lip, free Ole, and CS-Lip in EA.hy926 cells. ^†^Blank CS-Lip contained no oleuropein and was prepared at the dilution matching the corresponding CS-Ole-Lip concentration, equivalent to 0.05, 0.1, 0.2, and 0.4% *v/v* at 500, 1000, 2000, and 4000 ng/mL, respectively. Data are presented as mean ± SD from three independent experiments (*n* = 3), with four technical replicate wells per condition in each experiment. Each formulation series was analyzed independently using one-way ANOVA followed by Dunnett’s multiple-comparisons test against the corresponding untreated (zero-concentration) control. No comparisons were statistically significant (*p* > 0.05).

Notably, blank CS-Lip exhibited no significant cytotoxic effect at any tested dose, indicating cytocompatibility of the delivery vehicle under the specific in vitro conditions tested. This observation is consistent with earlier reports that CS-coated nanocarriers and liposomal systems exhibit minimal toxicity in endothelial and epithelial cell lines under selected in vitro conditions.^25^ The absence of toxicity in the blank formulation supports its potential as a safe platform for vascular-targeted delivery, although broader biocompatibility requires integrated in vivo safety, immune-response, and hemocompatibility evaluation.

Free Ole showed slightly lower mean viability than CS-Ole-Lip at some concentrations, but all mean values remained above 90%. Because the results do not show direct between-formulation significance, this pattern should not be interpreted as evidence that encapsulation protected mitochondrial function.

Importantly, no dose-dependent decrease in viability was observed across the 500–4000 ng/mL range, suggesting that both free and encapsulated Ole maintain a favorable safety profile even at the higher tested concentrations. The ability to maintain >90% cell viability in endothelial cells is particularly important for formulations intended for vascular or circulatory system applications, where prolonged exposure to the vascular endothelium requires minimal cytotoxicity to ensure safety and therapeutic efficacy.

The concentration range evaluated (500–4000 ng/mL) was selected based on effective anti-inflammatory concentrations observed in preliminary experiments. Although all formulations showed >90% viability across this range, the narrow concentration window precludes determination of IC_50_ values. Expanded dose–response studies across a wider concentration range (e.g., up to 100 μg/mL) would provide a more complete cytotoxicity profile and define the therapeutic index of CS-Ole-Lip in endothelial cells.

### 3.8 CS-Ole-Lip Suppresses Serum MCP-1 and IL-6 in TNF-α-Induced Vascular Inflammation in Mice

The anti-inflammatory efficacy of CS-Ole-Lip was evaluated in vivo using a TNF-α-induced vascular inflammation mouse model. Serum levels of proinflammatory cytokines, monocyte chemoattractant protein-1 (MCP-1) and interleukin-6 (IL-6), were quantified by ELISA to assess the systemic inflammatory response following treatment (Figure 8). Treatment with free Ole (20 mg/kg) moderately reduced MCP-1 by 1.67-fold compared with the TNF-α group (*p* < 0.01). Although IL-6 levels were reduced by 3.50-fold, this difference was not statistically significant. CS-Ole-Lip at 20 mg/kg produced the strongest suppression of both cytokines, reducing MCP-1 by 2.45-fold (*p* < 0.0001) and IL-6 by 6.90-fold (*p* < 0.05) compared with the TNF-α group, nearly restoring cytokine levels to those observed in healthy controls. Low-dose CS-Ole-Lip (2 mg/kg) also retained substantial activity, reducing MCP-1 by 1.76-fold (*p* < 0.001) and IL-6 by 4.45-fold (*p* < 0.05) compared with the TNF-α group. Ole-Lip at 20 mg/kg reduced MCP-1 and IL-6 levels by 2.21-fold (*p* < 0.0001) and 6.51-fold (*p* < 0.05), respectively, relative to the TNF-α group.

**Figure 8:**
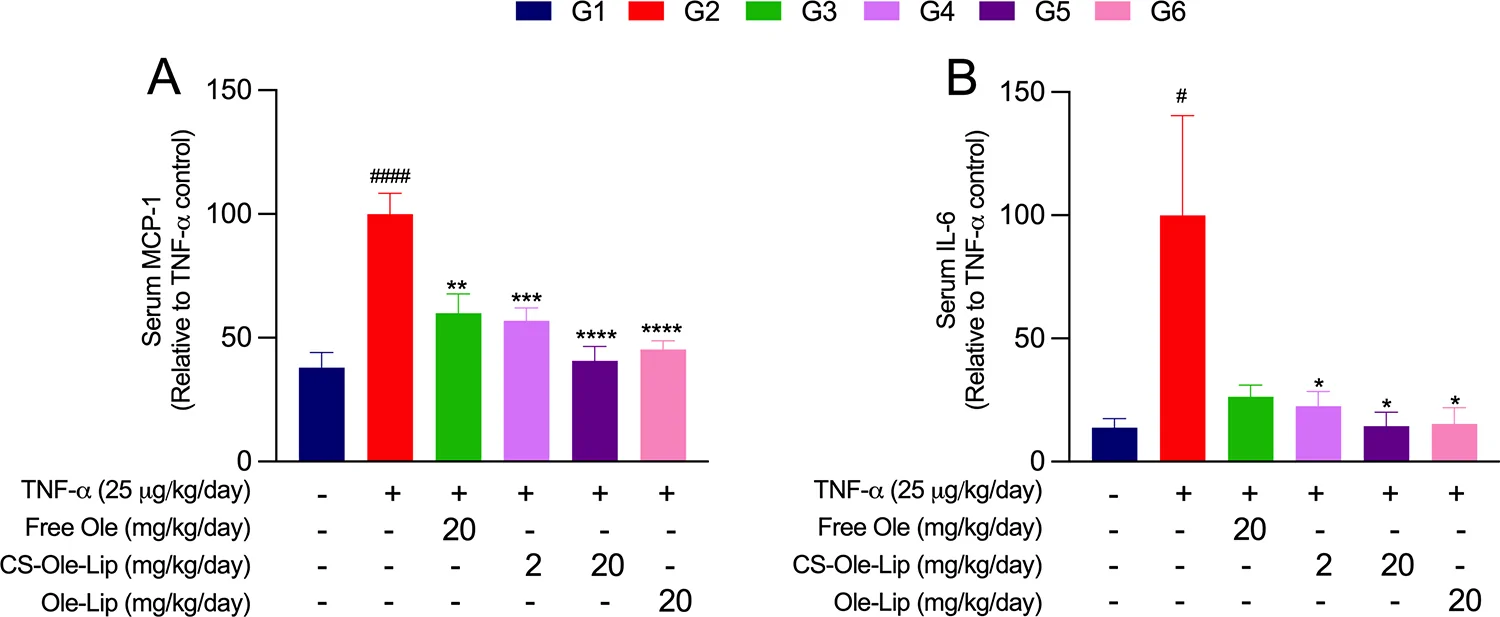
Serum levels of MCP-1 and IL-6 in the TNF-α-induced vascular inflammation model after treatment with free Ole (20 mg/kg), CS-Ole-Lip (2 mg/kg and 20 mg/kg), and Ole-Lip (20 mg/kg). #*p* < 0.05 and ####*p* < 0.0001 vs normal group (G1); *p < 0.05, **p < 0.01, ***p < 0.001, and ****p < 0.0001 vs TNF-α control group (G2). Data are presented as mean ± SEM (*n* = 5 mice per group). Comparisons were performed as G1 vs G2 and G2 vs each of G3–G6 by one-way ANOVA followed by Bonferroni-corrected pairwise comparisons.

Together, these results indicate that liposomal encapsulation and CS coating are associated with improved anti-inflammatory pharmacodynamic performance relative to free Ole. However, the present in vivo design did not include a chitosan-only, drug-free comparator group. Therefore, comparisons between Ole-Lip and CS-Ole-Lip reflect the combined effects of nanoliposomal encapsulation and CS coating, and the relative contribution of CS intrinsic antioxidant or anti-inflammatory activity cannot be fully isolated from carrier-related functions such as improved Ole retention, mucoadhesion, and oral exposure.

The numerically greater pharmacodynamic response of CS-Ole-Lip (20 mg/kg) relative to Ole-Lip (20 mg/kg) across both cytokines, 6.90-fold versus 6.51-fold IL-6 reduction and 2.45-fold versus 2.21-fold MCP-1 reduction, may be consistent with a CS coating contribution beyond simple liposomal encapsulation; however, no direct statistical comparison between the two liposomal groups was reported. The magnitude of this difference was modest at the 20 mg/kg dose, suggesting that both liposomal formulations delivered Ole in pharmacodynamically effective quantities and that the incremental benefit of CS coating at this dose may reflect marginal rather than dramatic differences in effective exposure. This pattern is qualitatively consistent with reports of CS-coated or CS-modified lipid nanocarriers improving antioxidant and anti-inflammatory activity relative to uncoated comparators, including soluble soybean polysaccharide/chitosan-encapsulated quercetin and chitosan-oligosaccharide-modified cordycepin nanoliposomes.^49,50^ The review of CS-coated liposome systems by Alcantara et al.^36^ identifies CS-mediated electrostatic interaction with negatively charged mucosal and cellular surfaces and prolonged gastrointestinal residence as primary mechanistic contributors to pharmacodynamic enhancement.

The most pharmacodynamically informative finding is the dose-sparing effect, where CS-Ole-Lip at 2 mg/kg, one-tenth of the 20 mg/kg dose evaluated in this study, achieved IL-6 suppression of 4.45-fold and MCP-1 suppression of 1.76-fold, both statistically significant relative to the TNF-α control group (*p* < 0.05) and numerically exceeding the activity of free Ole at 20 mg/kg (3.50-fold IL-6; 1.67-fold MCP-1). These pharmacodynamic contributors are mechanistically consistent with the physicochemical profile established in Sections 3.1 and 3.3: the sub-200 nm hydrodynamic diameter (168.73 nm) and narrow size distribution (PDI = 0.17) fall within the range reported to favor mucoadhesive retention and epithelial transcytosis, while the CS surface layer, evidenced by FTIR/XRD (Section 3.3), zeta potential shift relative to uncoated Ole-Lip, and the pH-dependent release barrier (Section 3.5), is compatible with prolonged intestinal residence and gradual Ole release that could affect systemic exposure. Although plasma Ole concentration–time profiling after oral gavage was not included in the present study, the marked cytokine suppression observed with low-dose CS-Ole-Lip provides pharmacodynamic evidence compatible with enhanced oral performance of the CS-coated formulation. The absence of pharmacokinetic data precludes a formal bioavailability claim; therefore, the dose-sparing effect is interpreted as a pharmacodynamic finding consistent with, but not proof of, CS-mediated enhancement of oral Ole delivery.

The suppression of serum MCP-1 and IL-6 is pharmacodynamically relevant to the vascular inflammatory context. MCP-1 (CCL2) is the principal chemoattractant driving monocyte recruitment to activated endothelium and subsequent foam cell formation, a rate-limiting early step in atherosclerotic plaque development,^3,4^ and IL-6 amplifies the systemic acute-phase response and drives STAT3-mediated endothelial dysfunction. While the present study did not directly measure endothelial NF-κB activation in vascular tissue, reduction of both markers by CS-Ole-Lip treatment thus addresses upstream drivers of endothelial inflammatory activation relevant to cardiovascular disease. This provides a coherent pharmacodynamic readout that aligns with the anti-inflammatory action of Ole attributed to NF-κB pathway inhibition in endothelial cells, reducing transcriptional drive for MCP-1 and IL-6 expression.^5,6^ Direct confirmation of the local endothelial pathway will require vascular tissue immunofluorescence for NF-κB p65 nuclear translocation, VCAM-1, and ICAM-1, together with in vitro NF-κB pathway assays in TNF-α-stimulated EA.hy926 cells. These experiments are therefore positioned as mechanistic validation studies in Section 3.10.

### 3.9 Safety Evaluation in Mice

To evaluate the systemic toxicity of the CS-Ole-Lip, serum biochemical markers of liver (ALT, AST) and kidney (BUN, CREA) function were quantified following treatment in the TNF-α-induced vascular inflammation mouse model. As shown in Figure 9, no statistically significant differences were observed in ALT, AST, CREA, and BUN levels among the treated groups and the healthy control group (*p* > 0.05; *n* = 3 per group). These findings indicate that administration of CS-Ole-Lip did not induce detectable hepatocellular injury or nephrotoxicity under the study conditions. To provide context for the effect of TNF-α challenge alone, ALT, AST, BUN, and CREA values for the G2 (TNF-α + PBS) group are reported in Supporting Information Table S3.

**Figure 9:**
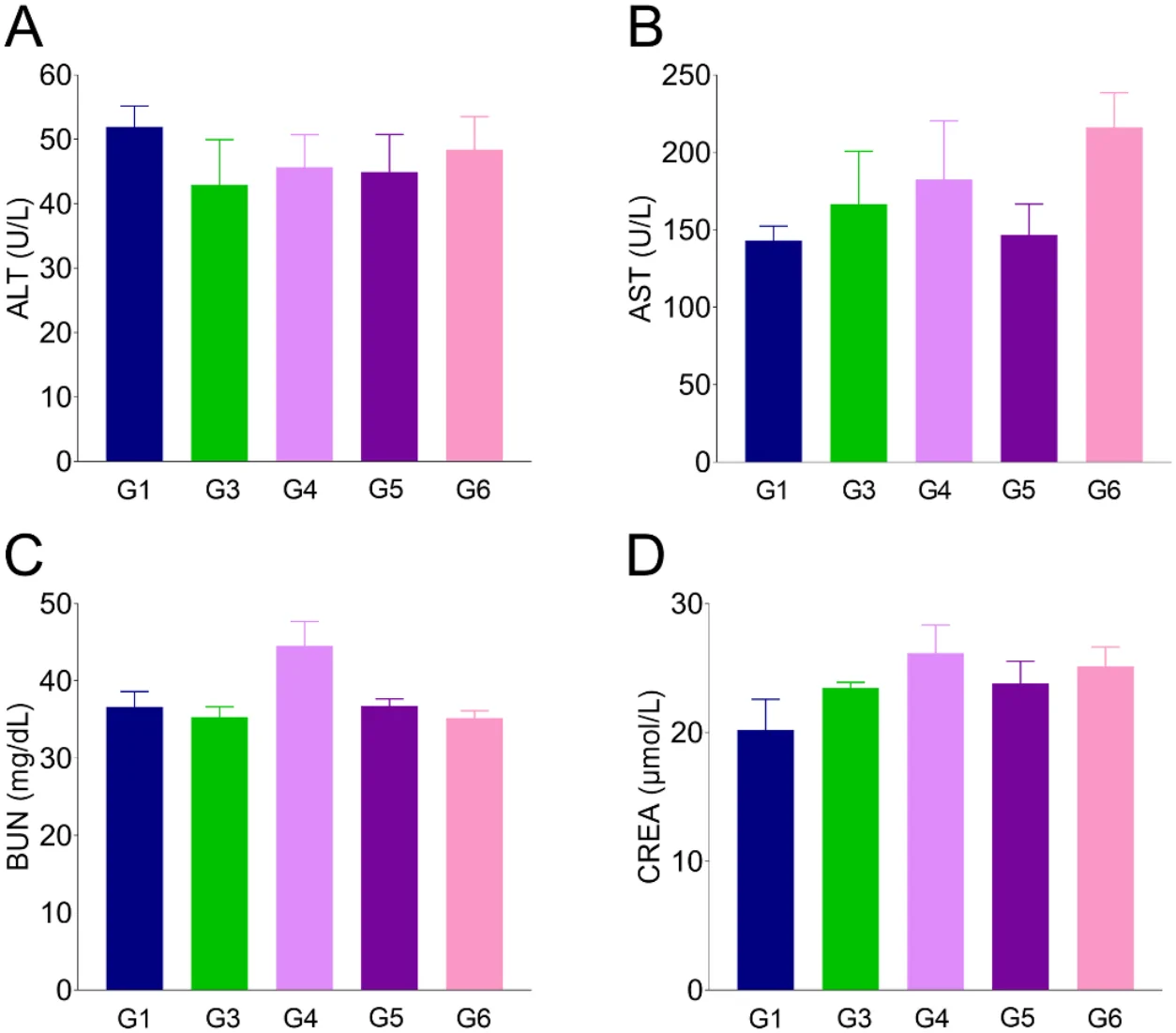
Serum biochemical markers of liver and kidney function, (A) ALT, (B) AST, (C) BUN, and (D) CREA after treatment. Groups: G1, normal (PBS only), G3, free Ole (20 mg/kg/day + TNF-α), G4, low-dose CS-Ole-Lip (2 mg/kg/day + TNF-α), G5, high-dose CS-Ole-Lip (20 mg/kg/day + TNF-α), G6, Ole-Lip (20 mg/kg/day + TNF-α). Data are presented as mean ± SEM (*n* = 3 mice per group). The same three mice were used for serum biochemical analysis and histopathological evaluation. No statistically significant differences were observed between G1 and the evaluated treatment groups (G3–G6) (*p* > 0.05). The G2 (PBS + TNF-α) biochemical marker values are reported separately in Supporting Information Table S3.

This systemic safety profile is consistent with prior reports demonstrating the biocompatibility of CS-based delivery systems and their minimal interaction with off-target organs at therapeutic doses.^47,51,52^

Histological evaluation was performed in G1, G3, and G5 to establish an organ-specific safety profile. The heart, liver, spleen, lungs, and kidneys were harvested and processed using hematoxylin and eosin (H&E) staining for microscopic observation (Figure 10). No overt signs of inflammation, necrosis, tissue degeneration, or leukocyte infiltration were observed across all examined organs in the CS-Ole-Lip-treated group. The liver exhibited well-preserved lobular architecture, while the kidney glomeruli and tubules appeared morphologically intact. Similarly, pulmonary alveolar structures, splenic white pulp, and cardiac muscle fibers remained unaffected. These histological findings reinforce the conclusion from the biochemical assays that CS-Ole-Lip possesses a favorable safety profile, at least under the dosage and treatment duration evaluated. Importantly, the histological normalcy of the kidneys and liver is particularly noteworthy given their critical roles in drug metabolism and excretion.

**Figure 10:**
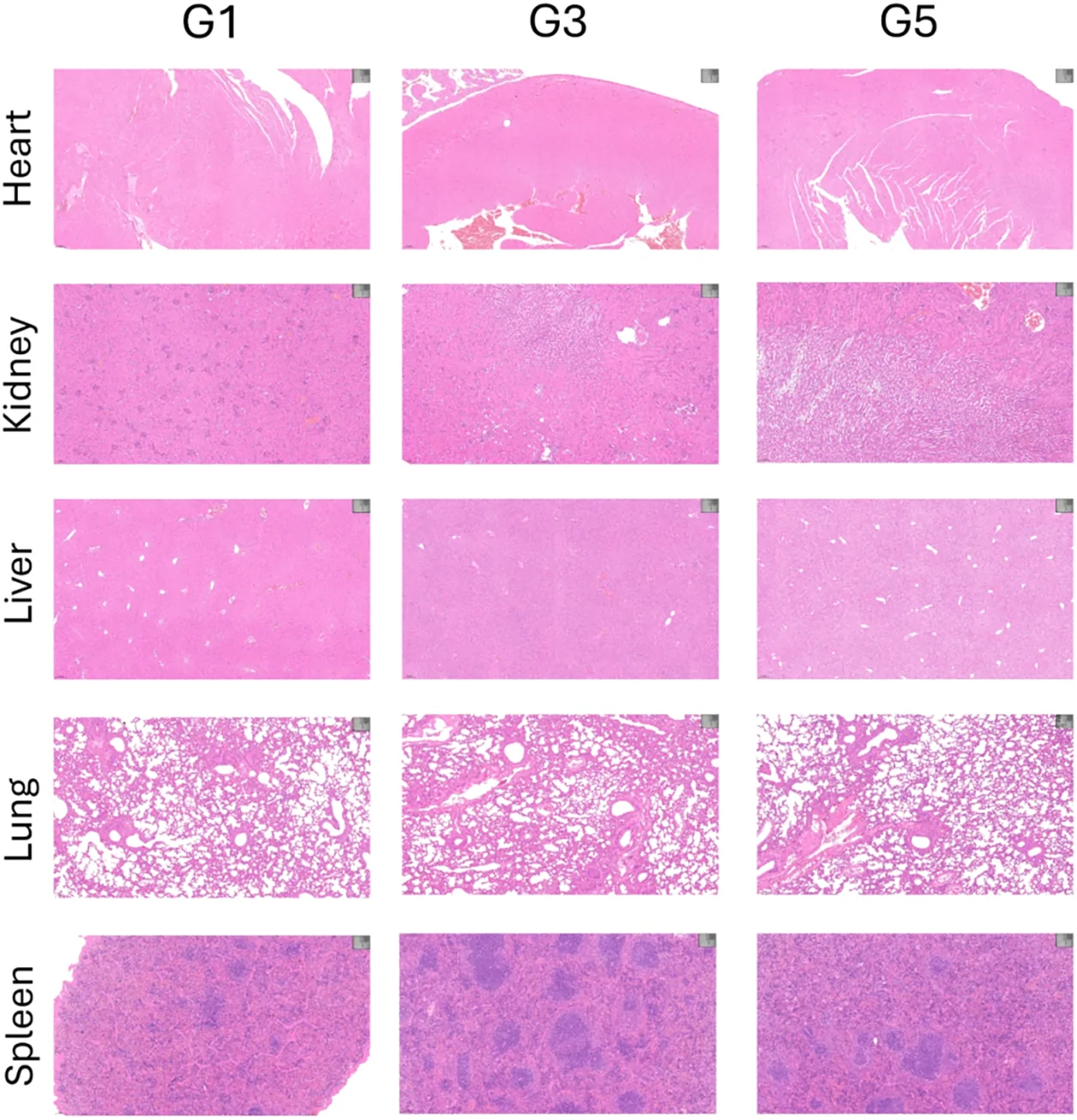
Representative H&E-stained images of major organs (heart, kidneys, liver, lungs, and spleen) from the normal control (G1), free Ole (G3), and highest-dose CS-Ole-Lip groups (G5) (*n* = 3 mice per group).

### 3.10 Limitations and Future Directions

The present study establishes the initial formulation, release, cellular interaction, and acute anti-inflammatory profile of CS-Ole-Lip. These findings provide a basis for the next stage of preclinical characterization, particularly in defining how chitosan coating influences oral exposure, endothelial delivery, and vascular inflammatory signaling. The current data support pharmacodynamic activity under controlled experimental conditions, while several additional studies can further refine the mechanistic and translational interpretation of the system.

A key next step is pharmacokinetic validation. The observed response pattern, in which CS-Ole-Lip at 2 mg/kg produced greater serum MCP-1 and IL-6 suppression than free Ole at 20 mg/kg, is consistent with enhanced oral performance after chitosan-coated liposomal delivery. Direct plasma Ole concentration–time profiling would strengthen this interpretation by determining whether the pharmacodynamic advantage is primarily exposure-driven. A future study comparing free Ole, Ole-Lip, and CS-Ole-Lip after oral gavage in C57BL/6 mice, with plasma sampling at 0, 0.5, 1, 2, 4, 6, 8, and 24 h and LC-MS/MS quantification of Ole, would allow calculation of Cmax, AUC_0–24_, Tmax, and relative oral bioavailability against an intravenous reference dose. In addition, the present in vivo design did not include a chitosan-only, drug-free comparator group. Because chitosan has documented intrinsic antioxidant and anti-inflammatory activity, part of the enhanced efficacy of CS-Ole-Lip relative to Ole-Lip could reflect a direct contribution of chitosan itself, in addition to CS-mediated improvements in Ole retention, mucoadhesion, and oral exposure. A future study incorporating a CS-only gavage arm, dose-matched to the chitosan content of the CS-Ole-Lip 20 mg/kg group, is needed to isolate this contribution.

The acute TNF-α-induced murine model provides a reproducible first-tier platform for evaluating anti-inflammatory nanoformulations because it generates defined cytokine end points through a controlled inflammatory stimulus. Within this scope, the model is appropriate for demonstrating systemic anti-inflammatory activity and comparing formulation-dependent pharmacodynamic responses. A logical extension would be validation in ApoE^–/–^ or LDLr^–/–^ mice maintained on a high-fat diet, where the performance of CS-Ole-Lip can be examined in the context of chronic lipid-driven vascular inflammation, plaque formation, and lesion progression.

The two-dose in vivo design was useful for identifying a preliminary pharmacodynamic dose-sparing pattern. Since CS-Ole-Lip at 2 mg/kg produced substantial cytokine suppression relative to free Ole at 20 mg/kg, a more detailed dose–response study would help define the magnitude of this effect quantitatively. A future five-dose comparison of CS-Ole-Lip and free Ole at 0.5, 2, 5, 10, and 20 mg/kg in the same TNF-α model would permit construction of dose–response curves for MCP-1 and IL-6, estimation of ED50 values, and calculation of a dose-sparing factor as the ED50 ratio between formulations. Future work extending the design space beyond the practically constrained boundaries identified here (Section 3.1) may further clarify whether higher LC is achievable without compromising particle size, PDI, coating integrity, or physical stability.

The cellular uptake experiment provides qualitative evidence of CS-Lip association with endothelial cells using a fluorescent FITC surrogate. This result supports the plausibility of chitosan-mediated cellular interaction, but quantitative confirmation would further define the contribution of the coating to nanocarrier uptake and Ole delivery. Flow cytometric analysis of fluorescently labeled CS-Lip cellular association, combined with LC-MS/MS quantification of intracellular Ole in EA.hy926 lysates after treatment with CS-Ole-Lip, Ole-Lip, and free Ole, would provide a stronger link between carrier uptake and payload delivery. The cellular uptake comparison was also limited to free FITC and FITC-loaded CS-Lip; inclusion of FITC-loaded uncoated liposomes in future experiments would more directly isolate the contribution of CS coating to endothelial uptake.

The serum cytokine results indicate suppression of MCP-1 and IL-6, both of which are relevant to vascular inflammatory activation. To connect this systemic response more directly with endothelial signaling, future studies should assess NF-κB p65 nuclear translocation, VCAM-1, and ICAM-1 expression in aortic sections using immunofluorescence. Complementary in vitro assays in TNF-α-stimulated EA.hy926 cells, such as NF-κB luciferase reporter analysis or nuclear p65 Western blotting, would clarify whether CS-Ole-Lip reduces inflammatory signaling through endothelial NF-κB pathway inhibition at matched Ole concentrations. The present study quantified only two serum cytokines, MCP-1 and IL-6, selected as representative markers of monocyte chemoattraction and the acute-phase inflammatory response, respectively. Additional mediators relevant to vascular inflammation and endothelial activation, including TNF-α, IL-1β, ICAM-1, VCAM-1, and C-reactive protein (CRP), were not measured and represent important extensions for future work.

The in vitro release study defined pH-dependent release behavior under controlled enzyme-free conditions, which was useful for isolating the influence of environmental pH on Ole release from CS-Ole-Lip. Further biorelevant testing can expand this release characterization by simulating gastrointestinal transit. A sequential model using simulated gastric fluid with pepsin at pH 1.2 for 2 h, followed by simulated intestinal fluid with pancreatin at pH 6.8 for 6 h, and subsequent incubation in PBS at pH 7.4 would determine whether the chitosan coating maintains its release-modulating function under digestive conditions, including enzyme-mediated effects on the liposomal bilayer. Formal saturation-solubility determinations of Ole in each release medium were not performed in this study; future work should confirm sink conditions experimentally by measuring Ole solubility in SGF, SIF, and pH 7.4 buffer containing 0.1% Tween 80. Future TEM characterization should also include desalting or buffer exchange before negative staining to avoid PBS salt-crystallization artifacts and provide clearer morphological confirmation.

Overall, the present findings position CS-Ole-Lip as an optimized oral liposomal formulation with sustained release behavior, endothelial cellular association, and acute systemic anti-inflammatory activity. The next phase of investigation should focus on pharmacokinetic profiling, quantitative uptake analysis, molecular pathway confirmation, dose–response modeling, and chronic atherosclerosis validation to define the formulation’s therapeutic potential with greater mechanistic and translational precision.

## 4 Conclusions

This study developed and optimized a CS-Ole-Lip system for oral anti-inflammatory delivery. The resulting uniform lipid–polymer nanocarrier, obtained using Box-Behnken design, yielded a well-characterized nanoscale formulation with high Ole EE and LC. The formulation further showed Fickian diffusion-governed sustained release behavior, refrigerated colloidal stability, and favorable in vitro cytocompatibility and in vivo safety profiles under the conditions evaluated.

In the TNF-α-induced murine vascular inflammation model, CS-Ole-Lip produced numerically greater serum IL-6 and MCP-1 suppression than free Ole and uncoated Ole-Lip at equivalent doses. Notably, CS-Ole-Lip at 2 mg/kg, one-tenth of the 20 mg/kg dose evaluated in this study, retained substantial cytokine suppression, suggesting a potential dose-sparing effect. These findings support the pharmacodynamic activity of the optimized CS-Ole-Lip system and support its potential as a stable oral nanocarrier with enhanced anti-inflammatory activity. Further plasma pharmacokinetic studies are required to determine whether this response is associated with improved oral Ole exposure, followed by expanded dose–response profiling, sequential biorelevant release testing, and validation in chronic atherosclerosis models.

## Supplementary Material



## Notes

Summary of the regression analyses of the responses (Table S1); release kinetic modeling of Ole from CS-Ole-Lip (Table S2); serum biochemical markers in the TNF-α-only group (Table S3) (DOCX)
