# Supplementary material for: Chitosan-Coated Oleuropein Nanoliposomes Enable Dose-Sparing Suppression of Serum Inflammatory Markers in a TNF-α-Induced Vascular Inflammation Model
Source: ACS Omega. 2026 Sep 23;11(39):59668–85. doi: 10.1021/acsomega.6c06263 (PMC13647880; doi:10.1021/acsomega.6c06263)
Supplement: Supplementary file 1 [file ao6c06263_si_001.docx]

Chitosan-Coated Oleuropein Nanoliposomes Enable Dose-Sparing Suppression of Serum Inflammatory Markers in a TNF-α-Induced Vascular Inflammation Model

*Roi Martin B. Pajimna^1,2,3^, John Wilfred T. Malabanan^3,4^, Nonthaneth Nalinratana^3,5^, Yilin Song^6^, Pranee Rojsitthisak^3,7^, Pornchai Rojsitthisak^3,8*^, Agnes L. Llamasares-Castillo^1,2,9*^*

^1^ The Graduate School, University of Santo Tomas, Manila 1015, Philippines.

^2^ Department of Pharmacy, Faculty of Pharmacy, University of Santo Tomas, Manila 1015, Philippines.

^3^	Center of Excellence in Natural Products for Ageing and Chronic Diseases, Chulalongkorn University, Bangkok 10330, Thailand.

^4^	Department of Pharmacy, College of Allied Health Sciences, Trinity University of Asia, Quezon City 1102, Philippines.

^5^	Department of Pharmacology and Physiology, Faculty of Pharmaceutical Sciences, Chulalongkorn University, Bangkok 10330, Thailand.

^6^	Department of Pharmaceutics, School of Pharmacy, Shenyang Pharmaceutical University, Shenyang, Liaoning 110016, China.

^7^	Metallurgy and Materials Science Research Institute, Chulalongkorn University, Bangkok 10330, Thailand.

^8^	Department of Food and Pharmaceutical Chemistry, Faculty of Pharmaceutical Sciences, Chulalongkorn University, Bangkok 10330, Thailand.

^9^	Research Center for the Natural and Applied Sciences, University of Santo Tomas, Manila 1015, Philippines.

* Corresponding authors.

Email: [pornchai.r@chula.ac.th](mailto:pornchai.r@chula.ac.th) (Po.R.); [alcastillo@ust.edu.ph](mailto:alcastillo@ust.edu.ph) (A.L.L.-C.)

**Supporting Information**

| **Table S1.** Summary of the regression analyses of the responses | | | | | |  |
| --- | --- | --- | --- | --- | --- | --- |
| **Response** | **Suggested Model** | ***p*-value, overall model** | ***p*-value, lack-of-fit** | **Adjusted R^2^** | **Predicted R^2^** | **Adequate precision** |
| Particle size | Quadratic | 0.0010 | 0.2313 | 0.9612 | 0.8091 | 19.1277 |
| PDI | Quadratic | 0.0003 | 0.4705 | 0.9688 | 0.8745 | 21.4212 |
| LC | Linear | 0.0001 | 0.1881 | 0.7938 | 0.6605 | 11.2595 |

**Table S2.** Release kinetic modeling of Ole from CS-Ole-Lip

| **Model** | **Buffer** | **Parameter** | **R^2^** | **AIC** | **MSC** |
| --- | --- | --- | --- | --- | --- |
| Zero-order  (F = k_0_ · t) | pH 1.2 | K_0_ = 0.7002 | 0.0186 | 108.7160 | -0.3291 |
|  | pH 6.8 | K_0_ = 0.6426 | -0.0741 | 107.5175 | -0.4303 |
|  | pH 7.4 | K_0_ = 0.7944 | 0.1429 | 110.0246 | -0.1856 |
| First-order  (F = 100 · [1 – e^-k^_1_^t^)]) | pH 1.2 | K_1_ = 0.0124 | 0.3071 | 104.1911 | 0.0190 |
|  | pH 6.8 | K_1_ = 0.0106 | 0.1947 | 103.7733 | -0.1423 |
|  | pH 7.4 | K_1_ = 0.0160 | 0.4791 | 103.5525 | 0.3123 |
| Higuchi  (F = k_H_ · t^0.5^) | pH 1.2 | K_H_ = 6.3462 | 0.7578 | 90.5274 | 1.0700 |
|  | pH 6.8 | K_H_ = 5.8682 | 0.7200 | 90.0395 | 0.9141 |
|  | pH 7.4 | K_H_ = 7.1155 | 0.8221 | 89.5863 | 1.3866 |
| Korsmeyer-Peppas  (F = k_KP_ · t^n^) | pH 1.2 | K_KP_ = 12.5586  n = 0.3204 | 0.8923 | 81.9922 | 1.7266 |
|  | pH 6.8 | K_KP_ = 12.2049  n = 0.3071 | 0.8835 | 80.6351 | 1.6375 |
|  | pH 7.4 | K_KP_ = 13.0980  n = 0.3396 | 0.9227 | 80.7428 | 2.0669 |
| Hixson-Crowell  (F = 100 · [1 - (1 – k_HC_ · t)^3^]) | pH 1.2 | K_HC_ = 0.0034 | 0.2079 | 105.9295 | -0.1147 |
|  | pH 6.8 | K_HC_ = 0.0030 | 0.1020 | 105.1894 | -0.2512 |
|  | pH 7.4 | K_HC_ = 0.0042 | 0.3665 | 106.0946 | 0.1168 |

**Table S3.** Serum biochemical markers in the TNF-α-only group (G2: TNF-α + PBS) expressed as mean ± SEM (n = 3).

| **Marker** | **Value** |
| --- | --- |
| ALT | 177.04 ± 37.15 U/L |
| AST | 257.77 ± 25.23 U/L |
| BUN | 38.30 ± 5.76 mg/dL |
| Creatinine | 25.85 ± 0.76 µmol/L |

**Note:** All three designated G2 serum samples were analyzed, with no exclusions. These values provide context for the serum biochemical safety panel because the main Figure 9 comparison includes G1 and formulation-treated groups (G3-G6). Formal statistical comparison of G2 against G1 was not part of the original analysis plan.
